# Hypoxia‐induced miR‐210‐3p expression in lung adenocarcinoma potentiates tumor development by regulating CCL2 mediated monocyte infiltration

**DOI:** 10.1002/1878-0261.13260

**Published:** 2024-03-22

**Authors:** Leena Arora, Debarun Patra, Soumyajit Roy, Sidhanta Nanda, Navneet Singh, Anita K. Verma, Anuradha Chakraborti, Suman Dasgupta, Durba Pal

**Affiliations:** ^1^ Department of Biomedical Engineering Indian Institute of Technology Ropar Punjab India; ^2^ Department of Pulmonary Medicine Postgraduate Institute of Medical Education & Research (PGIMER) Chandigarh India; ^3^ Department of Zoology, Kirori Mal College University of Delhi India; ^4^ Department of Experimental Medicine & Biotechnology Postgraduate Institute of Medical Education & Research (PGIMER) Chandigarh India; ^5^ Department of Molecular Biology & Biotechnology Tezpur University Assam India

**Keywords:** CCL2, HIF‐1Α, LUAD, miR‐210‐3p, monocyte infiltration

## Abstract

In most cancers, tumor hypoxia downregulates the expression of C‐C motif chemokine 2 (*CCL2*), and this downregulation has been implicated in monocyte infiltration and tumor progression; however, the molecular mechanism is not yet clear. We compared noncancerous and lung‐adenocarcinoma human samples for hypoxia‐inducible factor 1‐alpha (HIF‐1A), *microRNA‐210‐3p* (*mir‐210‐3p*), and CCL2 levels. Mechanistic studies were performed on lung adenocarcinoma cell lines and 3D tumor spheroids to understand the role of hypoxia‐induced miR‐210‐3p in the regulation of *CCL2* expression and macrophage polarization. HIF‐1Α stabilization increases miR‐210‐3p levels in lung adenocarcinoma and impairs monocyte infiltration by inhibiting *CCL2* expression. Mechanistically, miR‐210‐3p directly binds to the 3′untranslated region (UTR) of *CCL2* mRNA and silences it. Suppressing miR‐210‐3p substantially downregulates the effect of hypoxia on *CCL2* expression. Monocyte migration is significantly hampered in miR‐210‐3p mimic‐transfected *HIF‐1A* silenced cancer cells. In contrast, inhibition of miR‐210‐3p in *HIF‐1A*‐overexpressed cells markedly restored monocyte migration, highlighting a direct link between the miR‐210‐3p level and tumor monocyte burden. Moreover, miR‐210‐3p inhibition in 3D tumor spheroids promotes monocyte recruitment and skewing towards an antitumor M1 phenotype. Anti‐hsa‐miR‐210‐3p‐locked nucleic acid (LNA) delivery in a lung tumor xenograft zebrafish model caused tumor regression, suggesting that miR‐210‐3p could be a promising target for immunomodulatory therapeutic strategies against lung adenocarcinoma.

## Graphical abstract

Hypoxia‐associated stabilization of HIF‐1A markedly enhanced miR‐210‐3p expression, which directly targets CCL2 downregulation in lung adenocarcinoma, resulting in impaired monocyte infiltration and the stimulation of macrophage TAM polarization. Inhibition of miR‐210‐3p promoted monocyte recruitment and skewed towards an M1 phenotype in 3D tumor spheroids and a tumor xenograft model. Targeting miR‐210‐3p could be an effective therapeutic strategy for the management of lung adenocarcinoma.

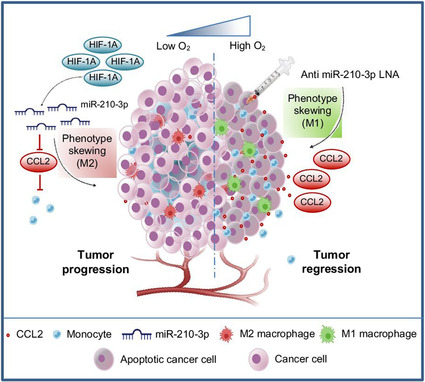

**Abbreviations**
3′UTR: 3′untranslated region
5‐Aza: 5‐Aza‐2′‐deoxycytidine
5mC: 5‐methylcytosine
AhR: aryl hydrocarbon receptor
ARNT: AhR nuclear translocator
CCL2: CC chemokine ligand 2
CCR2: C‐C chemokine receptor type 2
CD: cluster of differentiation
cDNA: complementary deoxyribonucleic acid
CoCl_2_
: cobalt chloride
DAPI: 4′,6‐diamidino‐2‐phenylindole
DNMT: DNA N‐methyl transferase
ELISA: enzyme‐linked immunosorbent assay
GAPDH: glyceraldehyde 3‐phosphate dehydrogenase
HIF‐1Α: hypoxia inducible factor‐1Α
HRE: hypoxia response element
HUVEC: human umbilical vein endothelial cell
Hypoxa miR: hypoxia‐associated microRNA
IL: interleukin
LNA: locked nucleic acid
LUAD: lung adenocarcinoma
miR‐210‐3p: microRNA‐210‐3p
MPEG1.1: macrophage expressed 1‐tandem duplicate 1
MPX: myeloid‐specific peroxidase
MTT: 3‐(4,5‐dimethylthiazol‐2‐yl)‐2,5‐diphenyltetrazolium bromide
NCCS: National Centre for Cell Science
NSCLC: non‐small‐cell lung cancer
OCT: optimal cutting temperature
ODDD: oxygen‐dependent death domain
OE: overexpression
PER: period circadian protein
PHD: prolyl hydroxylase domain
pVHL: protein Von Hippel–Lindau
RNAi: RNA interference
RT‐qPCR: real‐time quantitative PCR
SIM: single‐minded protein
TAM: tumor‐associated macrophage
TME: tumor microenvironment
U6 snRNA: U6 noncoding small nuclear RNA

## 1 Introduction

Lung cancer is the most prominent cause of cancer mortality worldwide, with non‐small cell lung cancer (NSCLC) accounting for around 85–90% of all lung cancers [1]. Lung adenocarcinoma (LUAD) is the most common form of NSCLC, accounting for 40% of all lung cancer subtypes, prevalent in nonsmokers and more common in women than men [2]. Hypoxia (low oxygen tension) is a well‐known factor contributing to advanced staged solid tumors, including LUAD [3, 4]. The rapid growth of tumor cells outgrows their surrounding vasculature, resulting in a drop in normal oxygen levels from 21% to 5.6% (oxygen level in normal peripheral tissues) and reach hypoxic levels of 1–2% in lung cancer [3, 4, 5]. Monocytes are the most abundant immune cell population infiltrating the tumor microenvironment of all solid tumors. The functions of these recruited monocytes/macrophages can be determined by a combination of factors, including the tumor type and stage, the degree of monocyte infiltration, and their functional polarization [6, 7]. Tumor‐associated macrophages (TAMs) are protumoral in nature and are involved in tumor initiation, progression, immune regulation, metastasis, and angiogenesis by the secretion of various antiinflammatory cytokines such as IL‐10, IL‐13, IL‐4, surface expression of arginase‐1, mannose receptor (MR, CD206), and scavenger receptors [8]. Within tumors, TAMs largely originate from progenitor cells (CCR2+ monocytes), which are recruited from the bone marrow by chemotactic cytokine ligand 2 (CCL2) [6, 9]. Tumor‐derived CCL2 is a potent monocyte‐chemotactic protein and its high level correlates with increased numbers of TAMs in tumor tissues [10, 11]. Previous studies indicated a conflicting role of hypoxia on *CCL2* expression. While chronic hypoxia downregulates the *CCL2* expression in ovarian cancer [12], and HUVEC [13], the *CCL2* upregulation was evident in gastric cancer [14], breast cancer cells [15], dermal fibroblasts [16], and primary mouse astrocytes [17]. This paradox is associated with the complex regulation of *CCL2* expression in response to hypoxic insult, as other epigenetic molecules also rule it. Thus, we tried to solve an important unanswered question: How chronic hypoxia affects the regulation of *CCL2* expression in LUAD and its involvement in lung cancer progression.

Recent reports suggest that hypoxia contributes to tumor progression by hypoxia‐inducible factor (HIF) stabilization, which induces changes in gene expression of cancer cells and the neighboring immune cells within the tumor microenvironment [18]. HIF is a heterodimeric transcription factor consisting of hypoxia‐inducible A‐subunits (HIF‐1A, ‐2A, and ‐3A) and constitutively expressed (nonoxygen responsive) HIF‐1B subunit of the PAS family (PER, AHR, ARNT, and SIM) [19]. Under normoxic conditions, cells continuously express and degrade HIF‐1Α protein by the action of prolyl hydroxylase domain (PHD) enzymes in the oxygen‐dependent death domain (ODDD) of α subunit. This hydroxylation of proline residues helps in the recognition and binding with E3 ubiquitin ligase complex, the von Hippel–Lindau tumor suppressor protein (pVHL), which leads to HIF‐A degradation. Under hypoxic conditions, suppression of propyl hydroxylation leads to the accumulation and nuclear localization of HIF‐A, which dimerizes with HIF‐1B. The heterodimer binds to the core pentanucleotide sequence (RCGTG) in the hypoxia response elements (HREs) of target genes and regulates the gene expression [19]. Hypoxia‐induced stabilization of the HIF‐1Α transcription factor accelerates tumor growth by upregulating the expression of various tumor supporting genes and multiple micro‐RNAs (miRNAs), including *mir‐210* [20]. miR‐210 is considered a predominant miRNA that increased under hypoxic conditions in various cancer types, and its transcriptional induction is dependent on HIF‐1Α stabilization [20, 21]. Moreover, miR‐210 is identified at the crossroad of hypoxia‐induced signaling pathways, as its expression level determines macrophage phenotypic skewing [22]. However, the role of miR‐210 on *CCL2* expression and its involvement in macrophage phenotype and migration has never been explored in the pathogenesis of LUAD, and thus requires intense investigation.

Hypoxia is known to stimulate the expression of *mir‐210‐3p*, as HIF‐1A directly binds to a hypoxia response element (HRE) on the proximal *mir‐210‐3p* promoter, located 400 bp upstream of the structure [23]. Thus, miR‐210‐3p stands out as a significantly upregulated “hypoxamiR” (hypoxia‐associated microRNA) [24], and we reasoned that it might play an essential role in regulating *CCL2* expression in the hypoxic tumor microenvironment, and thus affecting TAM abundance. This work provides the first evidence of the direct role of miR‐210‐3p on hypoxia‐induced *CCL2* downregulation resulting in impairment of monocyte infiltration and its conversion towards the M1 phenotype in LUAD cells, 3D lung tumor spheroids, and tumor xenograft zebrafish model. Together, these findings suggest that targeting miR‐210‐3p could be an effective therapeutic strategy for managing LUAD.

## 2 Materials and methods

### 2.1 Reagents and antibodies

All cell culture materials were either obtained from Gibco/Life Technologies (Grand Island, NY, USA) or Lonza (Walkersville, MD, USA). PBS/pU6‐HIF‐1ARNAi Plasmid, http://n2t.net/addgene: 21103; RRID: Addgene_21103 and pCAG‐HIF‐1A Plasmid, http://n2t.net/addgene: 21101; RRID: Addgene_21101 were a kind gift from C. Cepko (Addgene, Watertown, MA, USA). Wildtype CCL2‐3′UTR plasmid (HmiT016621‐MT06, miRNA 3′UTR target expression clone for Human CCL2 in pEZX‐MT06 vector with firefly luciferase reporter; GeneCopoeia, Rockville, MD, USA) was a kind gift from C. K. Sen (Indiana University School of Medicine, Indianapolis, IN, USA). miRIDIAN miRNA hairpin inhibitor negative control (cat. no. #IN‐001005‐01‐05), miRIDIAN miRNA hairpin mimic negative control (cat. no. #CN‐001000‐01‐05), miRIDIAN miRNA hsa‐miR‐210‐3p hairpin inhibitor (cat. no. #IH‐300565‐05‐0005), and miRNA hsa‐miR‐210‐3p hairpin mimic (cat. no. #C‐300565‐03‐0005) were purchased from GE Dharmacon (Lafayette, CO, USA). Custom MMU‐MIR‐210‐3p miRCURY LNA miRNA Power Inhibitor was purchased from Qiagen (Hilden, Germany). Antibodies against HIF‐1A (cat. no. #ab82832; RRID:AB_1860665 and cat. no. #ab16066; RRID:AB_302234) and β‐actin (cat. no. #ab119716; RRID:AB_10898702) were obtained from Abcam (Cambridge, MA, USA); antibody against 5‐methylcytosine (cat. no. #A‐1014, RRID:AB_2819207) was obtained from EpiGentek (Farmingdale, NY, USA) and antibody against CCL2 (cat. no. #sc‐32 771, RRID:AB_626820) was purchased from Santa Cruz Biotechnology (Santa Cruz, CA, USA). Anti‐VE‐cadherin (cat. no. #2158; RRID:AB_2077970) was purchased from Cell Signaling Technology (Danvers, MA, USA). Horseradish peroxidase‐conjugated antirabbit‐IgG (cat. no. #A9169, RRID:AB_258434), and antimouse‐IgG (cat. no. #A9044, RRID:AB_258431) were procured from Sigma‐Aldrich (St. Louis, MO, USA). NP40 lysis buffer (cat. no. #FNN0021) and Halt Protease and Phosphatase Inhibitor Cocktail (cat.no. #78440) were purchased from Invitrogen (Thermo‐Scientific, Grand Island, NY, USA). Clarity Western ECL Blotting Detection Reagent (cat. no. #1705060) and precision plus protein dual‐color standards (cat. no. #161–0374) were purchased from Bio‐Rad Laboratories (Hercules, CA, USA). We procured fluorochrome‐conjugated secondary antibodies, antimouse Alexa 488 (cat. no. #4408, RRID:AB_10694704), and antirabbit Alexa 594 (cat. no. #8889, RRID:AB_2716249) from Cell Signaling Technology; antimouse Alexa Fluor 594 (cat. no. #A‐11032, RRID:AB_2534091), and antirabbit Alexa Fluor 488 (cat. no. #A‐11034, RRID:AB_2576217) from Invitrogen (Thermo‐Scientific). U6 small nuclear RNA (snRNA) primer (cat. no. #4427975; ID: 001973) and hsa‐miR‐210 primer (cat. no. #4427975; ID: 000512) were obtained from Applied Biosystems (Foster City, CA, USA). The Dual‐Luciferase Reporter Assay Kit (cat. no. #E1910) was purchased from Promega (Madison, WI, USA). EpiQuik DNA Methyltransferase (DNMT) Activity/Inhibition Assay Kit (cat. no. #P‐3001‐1) was obtained from EpiGentek. 5‐Aza‐2′‐deoxycytidine (cat. no. #A3656) and Cobalt chloride (cat. no. #232696) were procured from Sigma‐Aldrich. HIF‐1A inhibitor‐PX‐478 (cat. no. #10005189) was purchased from Cayman Chemical (Ann Arbor, MI, USA).

### 2.2 Cell culture and treatments

Human lung adenocarcinoma A549 and NCI‐H460 cell lines were obtained from the National Centre for Cell Science (NCCS, Pune, India), and were maintained in Roswell Park Memorial Institute medium (RPMI) 1640 (cat. no. #A1049101; Gibco/Life Technologies) with 10% (v/v) Foetal Bovine Serum (FBS) (cat. no. #10082147; Gibco/Life Technologies) and 1% Penicillin–Streptomycin (cat. no. #15140122; Gibco/Life Technologies). Normal human bronchial epithelial cells BEAS‐2B and acute human monocytic leukemia cell line THP‐1 were a generous gift from A. K. Verma and R. Mukhopadhyay (Tezpur University, Assam, India), respectively, and were cultured in LHC‐9 medium (cat. no. #12680013; Gibco/Life Technologies) and RPMI 1640 medium, respectively. Hypoxia treatment was performed in HERACELL VIOS 160i incubator (Thermo Scientific) in 1% O_2_, and 5% CO_2_ levels at 37 °C unless stated otherwise. Cobalt chloride (CoCl_2_) was also used to induce hypoxia at a final concentration of 100 μm unless specified otherwise. For treatment with 5‐Aza‐2′‐deoxycytidine, A549 cells (0.3 × 10^6^ cells/well) were placed in a 6‐well plate and treated with 20 μm of 5‐Aza‐2′‐deoxycytidine for 48 h, and the media was changed every 24 h. Following incubation with 5‐Aza‐2′‐deoxycytidine, cells were exposed to normoxia or hypoxia for 24 h.

### 2.3 Human samples

Human lung tissue samples, pleural fluid, and blood serum were collected from patients with or without non‐small cell lung cancer (demographic details provided in Table S1) from the Postgraduate Institute of Medical Education and Research (PGIMER, Chandigarh, India). The Institute Ethics Committee (IEC), PGIMER, Chandigarh (Protocol No.: IEC‐12/2017–794), and the Institute Biosafety Committee (IBSC/1/2020/A/2) approved all human studies. The Declaration of Helsinki protocols were followed and informed written consent was obtained from all patients.

### 2.4 Zebrafish A549 tumor xenograft model and treatments

Zebrafish were maintained at 28 °C with a 14 h light / 10 h dark cycle in an automated stand‐alone housing system containing autoclaved, sterilized water containing a penicillin and streptomycin solution. Both males and females were used in this study. Animals were always handled and injected according to the principles of Good Animal Practice. Fish were fed three times daily with commercially available fish feed (MicroMac, Aqua World, India). The zebrafish lung cancer xenograft model was developed in the wildtype strain following a modified protocol described previously [25, 26]. For immune suppression, caerulomycin was administered in the intraperitoneal region of zebrafish at a dose of 100 mg·kg^−1^ body weight for immunosuppression 3 days prior to A549 cell transplantation and fish were maintained in water containing 1% penicillin and streptomycin. On day 3, 10^5^ cells/fish were suspended in phosphate‐buffered saline (PBS) and injected into the peritoneal cavity of the zebrafish using a 5 μL Hamilton syringe (Hamilton, NV, USA). Subsequently, fish were maintained in distilled water with 1% penicillin and streptomycin‐containing water for the next 14 days, allowing tumor xenograft development. On day 14, anti‐miR‐210 LNA (10 mg·kg^−1^) was administered intratumorally, and fish were sacrificed on day 3 and day 5 post‐treatment. Tumor tissue was collected, tumor size and tumor weight were measured, and photographed. The study protocol and procedures were approved by the University of Delhi, Institutional Animal Ethics Committee (Protocol No.: DU/KR/IAEC/ZF2021/1), and the Institute Biosafety Committee (IBSC/1/2020/A/2).

### 2.5 Transfection of HIF‐1ARNAi/overexpression plasmid and miR‐210‐3p mimic/inhibitor

For plasmid DNA transfection, Lipofectamine LTX reagent with PLUS reagent (cat. no. #15338100; Invitrogen, Thermo Fisher Scientific, Vantaa, Finland) was used following the manufacturer's instructions. Briefly, A549 cells (0.3 × 10^6^ cells/well) were seeded in a 6‐well plate in an antibiotic‐free complete growth medium for 24 h prior to transfection. For each well, 1 μg of plasmid DNA (PBS/pU6‐HIF‐1A RNAi or pCAG‐HIF‐1A) was added to 0.2 mL of OptiMEM serum‐free medium followed by the addition of 1 μL of PLUS reagent and incubated for 10 min at room temperature. Five microliter of Lipofectamine LTX reagent was then added to the diluted DNA sample and incubated for 25 min at room temperature. This reagent mixture was added to the cells containing 0.8 mL of complete growth medium and incubated for 24 h at 37 °C. Cells were then washed and a complete growth medium was added. After 48 h, cells were used for normoxia and hypoxia treatment. For transfection of miR‐210 mimic/inhibitor, Lipofectamine RNAi MAX transfection reagent (cat. no. #13778075; Invitrogen, Thermo Fisher Scientific) was used according to the manufacturer's protocol. Briefly, A549 cells (0.3 × 10^6^ cells/well) were seeded in a 6‐well plate in an antibiotic‐free complete growth medium for 24 h prior to transfection. For each well, 50 nm of control mimic/inhibitor or 100 nm of miR‐210‐3p mimic/inhibitor and 9 μL of Lipofectamine RNAi MAX reagent were added separately into 150 μL of OptiMEM medium. Both these solutions were mixed and incubated for 5 min. The transfection mixture was added to the cells containing 0.7 mL of complete growth medium and incubated for 48 h. After 48 h of transfection, cells were washed, a fresh complete growth medium was added, and used for normoxia or hypoxia treatment.

### 2.6 Immunoblotting

Control and treated cells were lysed in NP40 cell lysis buffer (cat. no. #FNN0021; Invitrogen) supplemented with the Halt protease and phosphatase inhibitor cocktail, centrifuged at 17 950 *g* for 10 min at 4 °C. Protein concentrations of cell lysates were determined by the BCA assay. Cell lysates (50 μg of protein) were resolved on 10% SDS/PAGE and transferred onto PVDF membranes (cat. no. #548IPVH00010; GE Healthcare Biosciences, Philadelphia, PA, USA) with the help of Turbo Blotting System (Bio‐Rad Laboratories). Membranes were first blocked with 5% bovine serum albumin (BSA) in TBS (Tris‐buffered saline) buffer for 1 h followed by an overnight incubation with primary antibodies (CCL2, 1 : 200 dilutions; HIF‐1A, 1 : 500 dilutions) in a rotating shaker at 4 °C. The membranes were then washed three times with TBST (TBS containing 0.1% Tween 20) buffer at 10‐min intervals and incubated with peroxidase‐conjugated specific secondary antibodies (1 : 2000 dilution) for 2 h at room temperature. Membranes were then washed three times with TBST at 10‐min intervals and subjected to Clarity Western ECL Substrate incubation for 5 min at room temperature. Protein bands were visualized in Chemidoc XRS+ System (Bio‐Rad Laboratories) using image lab Software.

### 2.7 Immunofluorescence staining

Cells were seeded at a density of 1.2 × 10^5^ cells on a sterile glass coverslip (22 mm) in 35‐mm cell culture plates and treated with normoxia or hypoxia conditions for 6 h. Cells were then fixed with 100% methanol for 5 min at −20 °C, washed with PBS, and permeabilized with 0.1% Triton X‐100 in PBS (v/v) for 10 min at room temperature. Cells were blocked with 1% BSA in PBS (w/v) containing 0.05% Tween (v/v) for 2 h at room temperature. Cells were then placed in a humidified chamber and incubated with primary antibodies (CCL2, 1 : 100 dilutions; HIF‐1A, 1 : 100 dilutions; and 5‐Methylcytosine, 1 : 200 dilutions) in 1% BSA in PBS containing 0.05% Tween overnight at 4 °C in a rotating platform. Cells were washed with ice‐cold PBS thrice for 5 min each, followed by the incubation with AlexaFluor 488‐tagged antimouse antibody (1 : 1000 dilutions) and AlexaFluor 594‐tagged antirabbit antibody (1 : 1000 dilutions) for 1 h at room temperature in the dark. Cells were then washed thrice for 5 min each with ice‐cold PBS and coverslips were mounted onto glass slides using VECTASHIELD HardSet Antifade Mounting Medium with DAPI (cat. no #H‐1500; Vector Laboratories, Burlingame, CA, USA). Cellular images were captured by an inverted fluorescence microscope (Leica DMi8, Leica‐Microsystems, Wetzlar, Germany) and image analysis was performed using lasx software (Leica‐Microsystems). Fluorescence intensity was quantified using imagej software (1.48v; NIH, Bethesda, MD, USA).

Immunofluorescence staining was also performed on tissue cryosections using specific antibodies. Briefly, OCT‐embedded tissue was cryo‐sectioned (10 μm), fixed with cold acetone, blocked with 5% BSA, and incubated overnight with specific primary antibodies. The Signal was visualized by subsequent incubation with fluorescence‐tagged appropriate secondary antibodies (AlexaFluor 488‐tagged antimouse, 1 : 1000 dilutions; AlexaFluor 594‐tagged antirabbit, 1 : 1000 dilutions) and counterstained with DAPI. Images were captured by a fluorescence microscope (Leica DMi8), and analysis was performed using lasx software.

### 2.8 Enzyme‐linked immunosorbent assay (ELISA)

CCL2 levels in human lung tissue, pleural fluid, serum, and the cell culture supernatant were quantified using LEGEND MAX Human MCP‐1/CCL2 ELISA Kit (cat. no. #438807; BioLegend, San Diego, CA, USA) following the manufacturer's instructions. Ten microgram protein was used to examine the CCL2 concentration from lung tissue samples. Briefly, the antibody‐coated well plate was washed with wash buffer followed by the addition of 50 μL of assay buffer along with 50 μL of diluted standards or samples in each well of the plate and incubated for 2 h at room temperature on a shaker. After 2 h, the content of the wells was discarded and washed. One hundred microliter of CCL2 detection antibody solution was added to each well and incubated for 1 h at room temperature on a shaker. Each well was washed thoroughly and 100 μL of Avidin‐HRP solution was added at 30‐min incubation at room temperature. The well content was then discarded and washed and 100 μL of substrate solution was added to each well at 15‐min incubation in the dark. On termination of incubations, 100 μL of stop solution was added to each well, and absorbance was measured at 450 and 570 nm on a microplate reader (Multiskan GO Microplate Spectrophotometer; Thermo Fisher Scientific). The absorbance at 570 nm was subtracted from the absorbance at 450 nm and the reading was plotted on the standard graph to calculate the CCL2 concentration in the samples.

### 2.9 RNA extraction and real‐time quantitative polymerase chain reaction (RT‐qPCR)

Total RNA was extracted from the cells of different incubations, human tissue, and zebrafish xenograft samples using Trizol reagent (cat. no. #15596018; Invitrogen) according to the manufacturer's instructions. RNA quality was measured using NanoDrop One/One Microvolume UV–Vis spectrophotometer (Thermo Fisher Scientific) and treated with DNase I. For mRNA expression analysis, cDNA was prepared from 500 ng of total RNA using iScript cDNA synthesis kit (cat. no. #1708891; Bio‐Rad Laboratories) following the manufacturer's guidelines. PowerUp SYBR Green Master Mix qPCR (2×) Universal was used to perform RT‐qPCR analysis in a Quant‐Studio 5 Real‐Time PCR System (Applied Biosystems) to quantify the relative mRNA expression level using gene‐specific primers. After the final extension, a melting curve analysis was performed to ensure the specificity of the products. Data were normalized to the expression of the *GAPDH* or *β‐Actin* reference gene. For miRNA expression analysis, 100 ng of total RNA was isolated from control and treated cells using miRVana miRNA isolation kit (cat. no. #AM1560; Ambion, Thermo Fisher Scientific, Austin, TX, USA), and specific miRNA expression was analyzed using the TaqMan miRNA RT Kit (cat. no. #4366596; Applied Biosystems, Thermo Fisher Scientific) followed by the TaqMan Universal PCR Master Mix (cat. no. #4324018; Applied Biosystems, Thermo Fisher Scientific). U6 snRNA was simultaneously amplified in a separate reaction and used as a loading control. Primer sequences used for RT‐qPCR are listed in Table S2. Gene expression was quantified using the delta–delta Ct relative quantization method using *GAPDH/β‐Actin* and *U6 snRNA* as a normalization control for mRNA and miRNA, respectively, and represented as fold change.

### 2.10 CCL2‐3′UTR luciferase reporter assay

A549 cells (0.1 × 10^6^ cell/well) were seeded in a 12‐well plate and after 24 h, cells were transfected with 1 μg of wildtype human CCL2‐3′UTR plasmid (HmiT016621‐MT06) or mutated CCL2‐3′UTR plasmid for 48 h using Lipofectamine LTX/Plus Reagent and then exposed to 21% oxygen (normoxia) or 1% oxygen (hypoxia). On termination of incubations, cells were lysed and luciferase activity was determined using the Dual‐Luciferase Reporter Assay System (Promega) in GloMax Navigator Microplate Luminometer (Promega) following the manufacturer's protocol. Data normalization was achieved by cotransfecting cells with 100 ng of Renilla plasmid. Relative luciferase activity was plotted as a ratio of firefly to Renilla luciferase activity.

### 2.11 Cell viability assay

Cell viability was assessed by 3‐(4,5‐dimethylthiazol‐2‐yl)‐2,5‐diphenyltetrazolium bromide (MTT) assay. Briefly, A549 and NCI‐H460 cells (4 × 10^3^ cells/well) were seeded in 96‐well plates 24 h prior to treatment. Cells were then treated with different concentrations of CoCl_2_ for 24 h followed by incubation with 10 μL (5 mg·mL^−1^) of MTT solution for 4 h. Upon termination of incubations, media were discarded and 100 μL of acidic isopropanol was added and kept for 30 min at 37 °C to dissolve the formazan crystals. The absorbance was measured at 570 nm on a microplate reader (MultiskanGO Microplate Spectrophotometer; Thermo Fisher Scientific, Finland). The absorbance values were blanked against acidic isopropanol to calculate the percentage of cell viability and the absorbance values of cells kept in a culture medium were considered the control (100% cell viability).

### 2.12 Monocyte migration assay

Monocyte migration assay was performed using transwell inserts containing 0.4 μm pores. A549 cells (0.5 × 10^6^ cells/well) were seeded in the lower chamber of 24‐well plates and after 24 h, cells were transfected with HIF‐1A RNAi or HIF‐1A overexpression plasmid and/or miR‐210‐3p mimic or miR‐210‐3p inhibitor for 48 h. Cells were then treated with normoxic or hypoxic conditions for 24 h. The serum‐starved THP‐1 monocytes were placed in the upper chamber of 0.4 μm transwell inserts and allowed for their migration for 16 h. Recombinant CCL2 protein was used as a positive control. Using a cotton swab, the upper membrane surface of the inserts was gently scrubbed, and the migrated cells in the lower membrane surface were first fixed with 2.5% glutaraldehyde solution for 10 min and then stained with 0.5% crystal violet stain for 2 h. The migrated cell numbers in each membrane were calculated for at least five random fields and cell images were captured using a microscope (Leica DMi8) at a magnification of 200×.

### 2.13 DNA methyltransferase assay

Nuclear proteins were extracted from control and treated A549 cells using the EpiQuik Nuclear Extraction Kit (cat. no. #OP‐0022; EpiGentek) following the manufacturer's instructions and the protein concentration was determined by BCA assay. DNMT activity was measured in the nuclear extract of A549 cells using the EpiQuik DNMT Activity/Inhibition Assay Kit (cat. no. #P3001‐1; EpiGentek) following the manufacturer's protocol. Briefly, 4 μg of nuclear protein was added to each well of 96‐well plates precoated with a DNMT substrate and incubated for 90 min at 37 °C. The well content was discarded, washed thoroughly with wash buffer, and incubated with the 50 μL of capture antibody solution for 60 min at room temperature on a shaker. After removing the well content, each well was washed with wash buffer and incubated with 50 μL of detection antibody solution for 30 min at room temperature on a shaker. The well content was then removed and washed thoroughly with wash buffer. One hundred microliter of developer solution was added to each well and incubated for 10 min at room temperature in the dark. On termination of incubations, 50 μL of stop solution was added to each well and the absorbance was measured at 450 nm on a microplate reader (Multiskan GO Microplate Spectrophotometer; Thermo Fisher Scientific, Finland) which was used to calculate the DNMT activity [DNMT activity (OD·h^−1^·mg^−1^) = (Sample OD − Blank OD/Protein amount (μg) × h) × 1000].

### 2.14 Lung tumor 3D spheroid development and monocyte infiltration

A549 and NCI‐H640 lung tumor 3D spheroids along with BEAS‐2B normal lung epithelial cells 3D spheroids were prepared by following the hanging drop plate method [27]. Briefly, A549, NCI‐H640, and BEAS‐2B cells (10 000 in number) were separately seeded per drop, and plates were maintained at 37 °C in a humidified incubator with 5% CO_2_ environment for 4 days to allow the spheroids to form. Cells were routinely observed under a microscope to examine cell aggregation and proliferation. The growth medium was then changed on alternate days by replacing 10 μL medium with 10 μL fresh culture medium for 10 days. On day 10, THP‐1 monocytes (1 × 10^3^ cells/spheroid) were added to the culture medium, and exposed to normoxia or hypoxia conditions for 24 h [28]. In a separate experiment, A549 and NCI‐H640 tumor spheroids were transfected with control or miR‐210‐3p inhibitor and after 48 h of transfection, THP‐1 monocytes (1 × 10^3^ cells/spheroid) were added to the culture medium for 24 h. On termination of incubations, cell spheroids were collected, stained with anti‐CD11b antibody, and the number of infiltrated monocytes was analyzed using a flow cytometer (BD Accuri C6 Plus Flow Cytometer; BD Biosciences, Franklin Lakes, NJ, USA).

### 2.15 Site‐directed mutagenesis assay

Wildtype CCL2‐3′UTR plasmid construct was used as a template for the generation of mutated CCL2‐3′UTR plasmid by using QuickChange Lightning Multi Site‐Directed Mutagenesis Kit (cat. no. #210515; Agilent Technologies, Santa Clara, CA, USA) following the manufacturer's instructions. Primers used to generate the mutated CCL2‐3′UTR plasmid were designed with the help of QuikChange Primer Design Program (www.agilent.com/genomics/qcpd). Primer sequences used for the generation of mutated CCL2‐3′UTR plasmid are listed in Table S1.

### 2.16 Statistical analysis

All data analyses were performed using graphpad prism software (v. 8.0; GraphPad Software, La Jolla, CA, USA). All experiments were performed at least three times unless otherwise indicated. Data are presented as mean ± SD. Statistical significance was analyzed by Student's *t*‐test or ANOVA with Tukey's multiple comparison test. A level of *P* < 0.05 was considered significant.

## 3 Results

### 3.1 Hypoxic lung adenocarcinoma microenvironment is associated with the lower level of CCL2 and higher expression of miR‐210‐3p

**Fig. 1:**
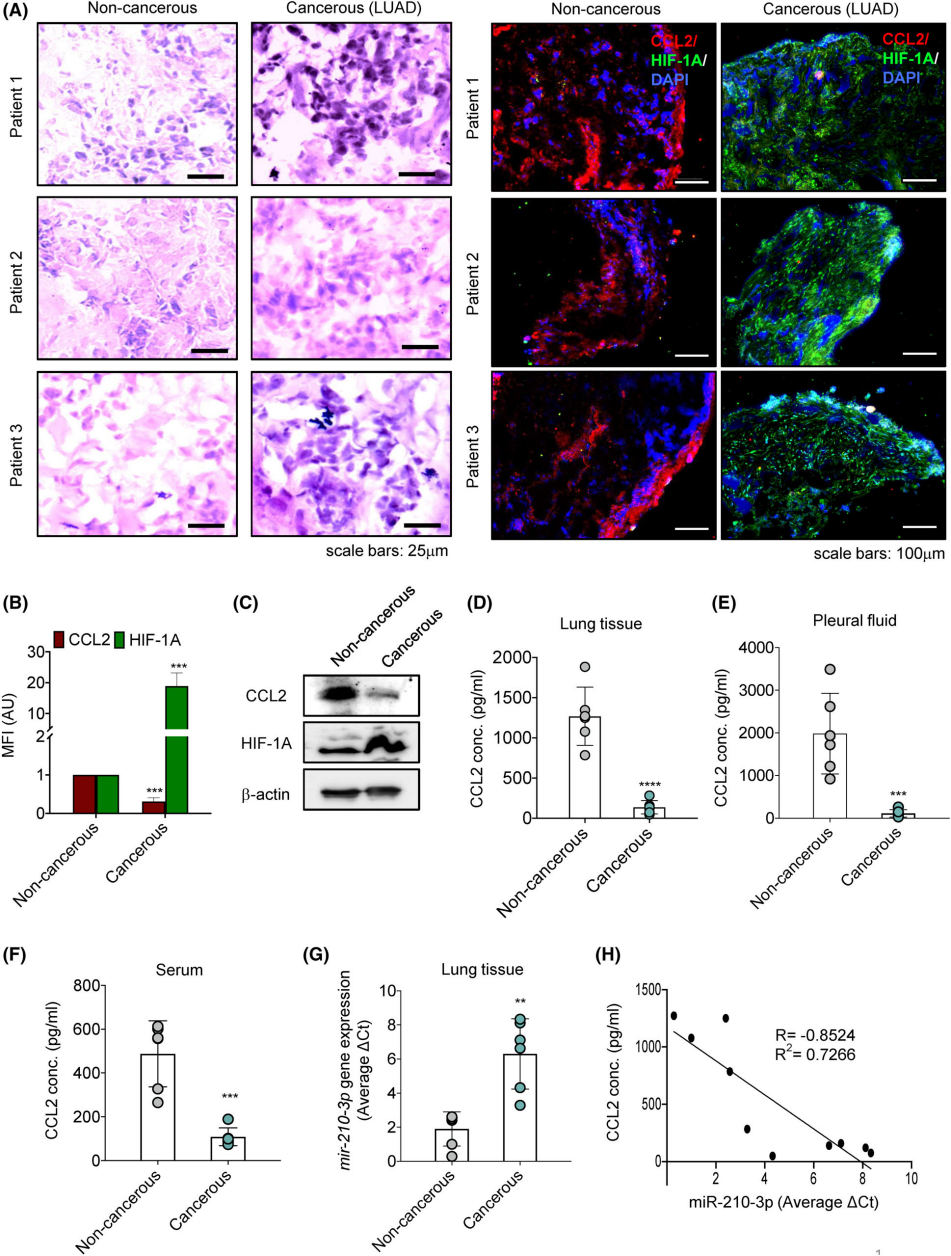
Hypoxic tumor microenvironment alters the expression of CCL2 and *mir‐210‐3p* in lung adenocarcinoma. (A) Hematoxylin and eosin (H&E) (left panel) and immunofluorescence (right panel) staining images of noncancerous (*n* = 3) and cancerous (lung adenocarcinoma, LUAD) (*n* = 3) tissue sections for visualization of morphology along with CCL2 (red) and HIF‐1A (green) protein levels. Nuclei were counterstained with DAPI (blue). Scale bars: 15 μm for H&E staining and 100 μm for immunofluorescence staining. Three images (*N* = 3) were taken from each sample. (B) The mean fluorescence intensity (MFI) of CCL2 and HIF‐1A levels in noncancerous and cancerous (LUAD) tissue sections of these patients were quantified as relative values of three patients (*n* = 3) and three values per sample (*N* = 3) using the imagej program and represented as a bar diagram. (C) Immunoblots showing the abundance of CCL2 and HIF‐1A proteins in noncancerous and cancerous (LUAD) tissue samples (*n* = 1). β‐Actin served as a loading control. (D–F) The concentration of CCL2 in 10 μg of tissue lysate (D), pleural fluid (E), and serum (F) of noncancerous (*n* = 6) and cancerous (LUAD) (*n* = 6) patients' samples were determined through ELISA. (G) RT‐qPCR analysis showing *mir‐210‐3p* gene expression in noncancerous (*n* = 6) and cancerous (LUAD) (*n* = 6) tissue samples. U6 snRNA was used as an internal reference control for miRNA normalization. (H) Inverse correlation between miR‐210‐3p mRNA and CCL2 protein expression in noncancerous and cancerous (LUAD) patient samples (*P* = 0.0017, Pearson *r* = −0.8524, *r* ^2^ = 0.7266). Data represent mean ± SD. Statistical significance was analyzed by Student's *t*‐test. **P < 0.01, ***P < 0.001, and ****P < 0.0001 vs noncancerous.

To examine the effect of hypoxia on CCL2 expression, we performed immunofluorescence staining of HIF‐1Α and CCL2 proteins in noncancerous and cancerous lung adenocarcinoma tissue sections. We have found that the increased abundance of HIF‐1Α coincided with the subdued amount of CCL2 in the cancerous tissue sections compared to noncancerous samples (Fig. 1A–C and Fig. S1A–E). The lower level of CCL2 was also evident in the tissue lysates (Fig. 1D), pleural fluid (Fig. 1E and Fig. S1F), and serum sample (Fig. 1F) of lung adenocarcinoma patients. Since hypoxia‐induced miRNA‐210‐3p is known to regulate the expression of multiple genes related to tumor progression, metabolism, and angiogenesis along with its crucial role in inflammation [20, 23], we were therefore interested in analyzing the *mir‐210‐3p* expression in lung adenocarcinoma. Interestingly, we noticed a significant upregulation of *mir‐210‐3p* gene expression in the lung adenocarcinoma tissue compared to noncancerous tissue (Fig. 1G). A negative correlation between *mir‐210‐3p* expression and CCL2 levels in human lung adenocarcinoma patients (Fig. 1H) incites further investigation of the role of miR210‐3p in CCL2 regulation in hypoxic tumors.

### 3.2 miR‐210‐3p silences *CCL2* expression by direct binding to its 3′UTR region in HIF‐1A stabilized lung adenocarcinoma

**Fig. 2:**
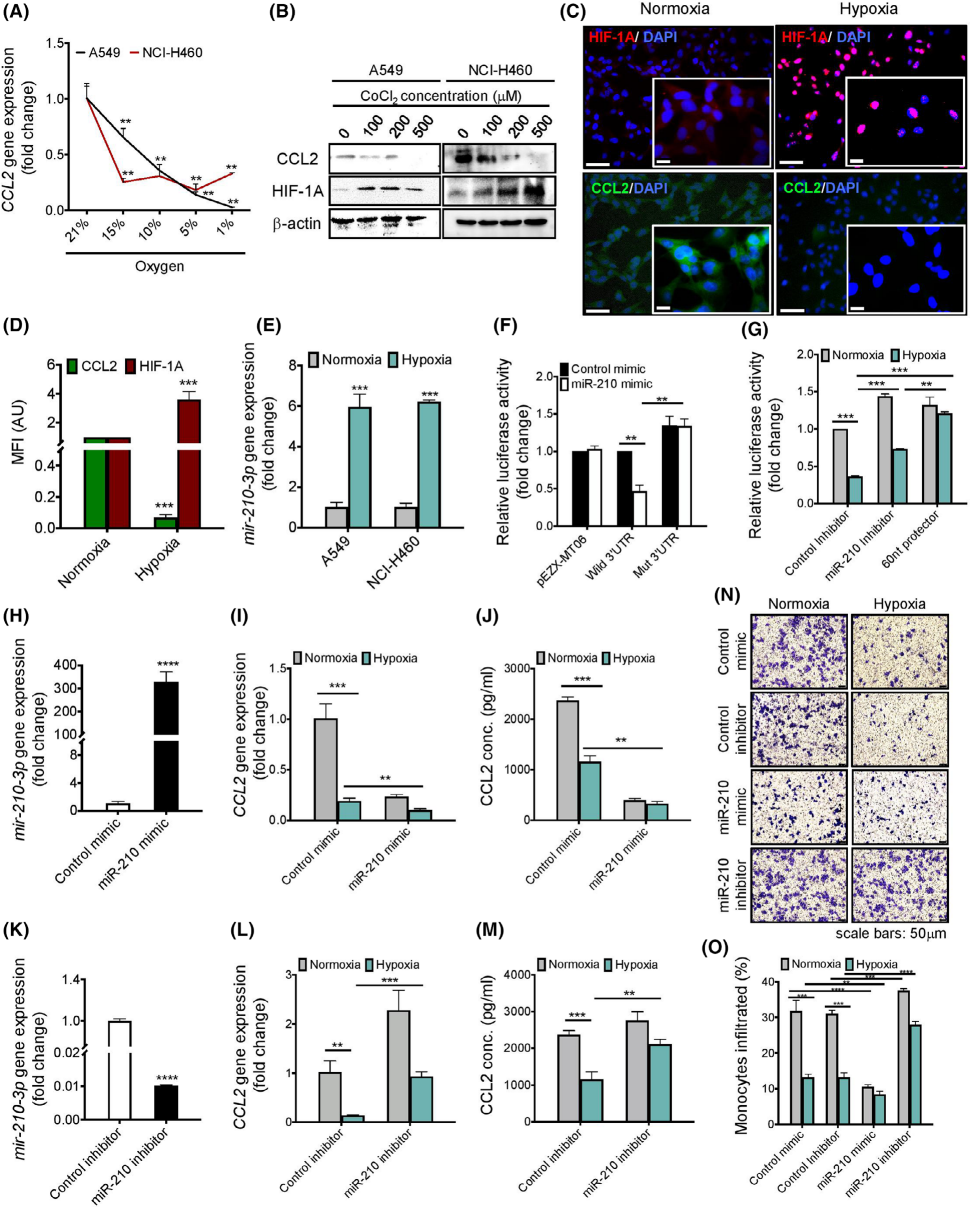
Hypoxia‐induced miR‐210‐3p directly inhibits *CCL2* expression attenuating monocyte infiltration in lung adenocarcinoma cell lines. (A) RT‐qPCR analysis indicating relative abundance of *CCL2* mRNA levels in A549 and NCI‐H460 cell lines incubated with indicated levels of oxygen for 24 h. *GAPDH* was used as an internal loading control for normalization. Data represented as mean ± SD of three independent experiments. Statistical significance was analyzed by Student's *t*‐test, **P < 0.01 vs 21% oxygen. (B) Immunoblots showing the abundance of CCL2 and HIF‐1A proteins in A549 and NCI‐H460 cells treated with indicated concentrations of CoCl_2_. β‐Actin was served as a loading control. (C,D) Representative immunofluorescence images (20×, scale bars: 50 μm and 63×, scale bars: 10 μm) (C) and their quantifications (D) showing HIF‐1A (red) and CCL2 (green) levels in A549 cells incubated under normoxic and hypoxic conditions for 24 h, respectively. Data represent the mean ± SD of three independent experiments. Statistical significance was analyzed by Student's *t*‐test, ***P < 0.001 vs normoxia. (E) RT‐qPCR analysis showing *mir‐210‐3p* gene expression in A549 and NCI‐H460 cells incubated in normoxic and hypoxic conditions for 24 h. *U6 snRNA* was used as an internal reference control for miRNA normalization. Data represented as mean ± SD of three independent experiments. Statistical significance was analyzed by Student's *t*‐test, ***P < 0.001 vs normoxia. (F,G) miR target reporter luciferase assay showing relative luciferase activity in control vector, wildtype, and mutated CCL2‐3′UTR transfected A549 cells treated with control mimic or miR‐210‐3p mimic (F) or in wildtype CCL2‐3′UTR transfected A549 cells treated with miR‐210‐3p inhibitor or 60 nt long miR‐210‐3p target protector under normoxic and hypoxic conditions for 24 h (G). Data represent the mean ± SD of three independent experiments. Statistical significance was analyzed by two‐way ANOVA, ***P < 0.001, **P < 0.01. (H) RT‐qPCR analysis of *mir‐210‐3p* gene expression in A549 cells transfected with control mimic or miR‐210‐3p mimic. *U6 snRNA* was used as an internal reference control. Data represent the mean ± SD of three independent experiments. Statistical significance was analyzed by Student's *t*‐test, ****P < 0.0001 vs control mimic. (I,J) RT‐qPCR analysis of *CCL2* gene expression (I) and ELISA showing CCL2 and HIF‐1A protein abundance (J) in control mimic or miR‐210‐3p mimic transfected A549 cells incubated with normoxic or hypoxic conditions for 24 h. *GAPDH* was used as a loading control for RT‐qPCR. Data represented as mean ± SD of three independent experiments. Statistical significance was analyzed by two‐way ANOVA, ***P < 0.001, **P < 0.01. (K) RT‐qPCR analysis of *mir‐210‐3p* gene expression in A549 cells transfected with control inhibitor or miR‐210‐3p inhibitor. *U6 snRNA* was used as an internal reference control. Data represent the mean ± SD of three independent experiments. Statistical significance was analyzed by Student's *t*‐test, ****P < 0.0001 vs control inhibitor. (L,M) RT‐qPCR analysis of *CCL2* gene expression (L) and ELISA showing CCL2 protein abundance (M) in control inhibitor or miR‐210‐3p inhibitor transfected A549 cells treated with normoxic or hypoxic conditions for 24 h. *GAPDH* was used as a loading control for RT‐qPCR. Data represented as mean ± SD of three independent experiments. Statistical significance was analyzed by two‐way ANOVA, ***P < 0.001, **P < 0.01. (N,O) Representative images (N) and their quantifications (O) of THP‐1 monocytes migration from the upper surface to lower surface of transwell insert placed on the well containing A549 cells transfected without or with miR‐210‐3p mimic or miR‐210‐3p inhibitor and exposed to normoxic or hypoxic conditions for 16 h. Scale bars: 50 μm. Data represent the mean ± SD of three independent experiments. Statistical significance was analyzed by two‐way ANOVA, ****P < 0.0001, ***P < 0.001, **P < 0.01.

We cultured two different lung adenocarcinoma cell lines (NCI‐H460 and A549) at different oxygen levels (21–1% oxygen) for 24 h. We noticed a significant downregulation of *CCL2* mRNA and its protein expression both in NCI‐H460 and A549 cells as oxygen tension was lowered compared to the normoxic environment (Fig. 2A and Fig. S2A). We found a significant downregulation in *CCL2* expression under nonhypoxic conditions (15–10% O_2_), which could possibly due to the basal expression of HIF‐1A in normal conditions (Fig. 2A). Also, with the increasing concentrations of CoCl_2_, a well‐known inducer of cellular hypoxia, a concentration‐dependent downregulation of CCL2 and concurrent enhancement of HIF‐1A were observed in A549 cells (Fig. 2B and Fig. S2B). Time‐dependent downregulation of the *CCL2* gene expression was also found in A549 cells treated with 1% oxygen (Fig. S2C). It should be noted here that both *CCL2* mRNA and protein levels were significantly downregulated as HIF‐1A stabilized (Fig. 2C,D and Fig. S2D–F). Moreover, we also analyzed the cell viability in response to different concentrations of CoCl_2_ to nullify the possibility of cell death. We have not observed any significant cytotoxicity with 100 μm CoCl_2_ concentration in either A549 and NCI‐H460 cell lines (Fig. S2G), and hence this concentration was chosen to perform further studies. Interestingly, both NCI‐H460 and A549 cells also showed a massive induction of *mir‐210‐3p* gene expression in a hypoxic environment compared to normoxia (Fig. 2E).

To investigate the possible interrelationship between miR‐210‐3p and *CCL2*, we performed *in‐silico* analyses to identify potentially relevant miR‐210‐3p target genes using the RNAhybrid miRNA target prediction algorithm (Bielefeld University BioInformatics Server, Germany). Intriguingly, we noticed that the 3′untranslated regions (3′UTRs) of the *CCL2* mRNA contained several putative binding sites for miR‐210‐3p; however, the major binding site for miR‐210‐3p lies between 579 and 586 nucleotides (ACCACAG) in the 3′UTR region of *CCL2* mRNA (Fig. S2H). To confirm the specificity of miR‐210‐3p direct binding to the *CCL2*‐3′UTR region, we performed an miR target reporter luciferase assay using wildtype and mutated *CCL2*‐3′UTR (579–586) along with control or miR‐210‐3p mimic (Fig. 2F and Fig. S2I). Delivery of the miR‐210‐3p mimic significantly suppressed *CCL2*‐3′UTR reporter luciferase activity in A549 cells transfected with wildtype *CCL2*‐3′UTR‐luc as compared to the control vector; however, such an effect was abrogated in A549 cells transduced with a mutated *CCL2‐*3′UTR‐luc plasmid (Fig. 2F). To confirm a direct binding of miR‐210‐3p on *CCL2‐*3′UTR in a hypoxic pathophysiological state, we delivered either a 60‐nucleotide long miR‐210‐3p target protector (Fig. S2I) or miR‐210‐3p inhibitor in wildtype *CCL2‐*3′UTR‐luc plasmid transfected cells and exposed them to normoxia or hypoxia conditions (Fig. 2G). Delivery of miR‐210‐3p target protector or miR‐210‐3p inhibitor significantly prevented hypoxia‐induced interaction of miR‐210‐3p and *CCL2‐*3′UTR, as indicated by the rescue of *CCL2‐*3′UTR luciferase activity. Moreover, transfection of the target protector in A549 cells significantly prevented miR‐210‐3p interaction with *CCL2‐*3′UTR in the presence of miR‐210‐3p mimic (Fig. S2J). These results clearly suggest a direct contact between miR‐210‐3p and *CCL2‐*3′UTR under a hypoxic state and that could be responsible for hypoxia‐associated downregulation of *CCL2* expression. To examine the direct impact of miR‐210‐3p on *CCL2* expression, we treated the A549 cells with miR‐210‐3p mimic or inhibitor both in normoxic or hypoxic conditions. Application of the miR‐210‐3p mimic markedly impaired *CCL2* mRNA and its protein levels (Fig. 2H–J), whereas the miR‐210‐3p inhibitor treatment significantly increased *CCL2* expressions (Fig. 2K–M) in A549 lung cancer cells. These observations validate that *CCL2*, a member of the CC chemokine family, is subject to post‐transcriptional gene silencing by miR‐210‐3p in the hypoxic LUAD. Furthermore, miR‐210‐3p inhibitor transfected A549 cells caused profound monocyte migration in normoxic conditions; however, such an effect was significantly attenuated in a hypoxic environment (Fig. 2N,O). In contrast, miR‐210‐3p mimic transfected A549 cells restricted the monocyte migration both in normoxic and hypoxic environments (Fig. 2N,O). All these results indicate a direct involvement of miR‐210‐3p in hypoxia‐induced *CCL2* downregulation and the impairment of monocyte migration in LUAD.

### 3.3 HIF‐1A regulates miR‐210‐3p‐mediated *CCL2* inhibition and monocyte infiltration in hypoxic lung adenocarcinoma

**Fig. 3:**
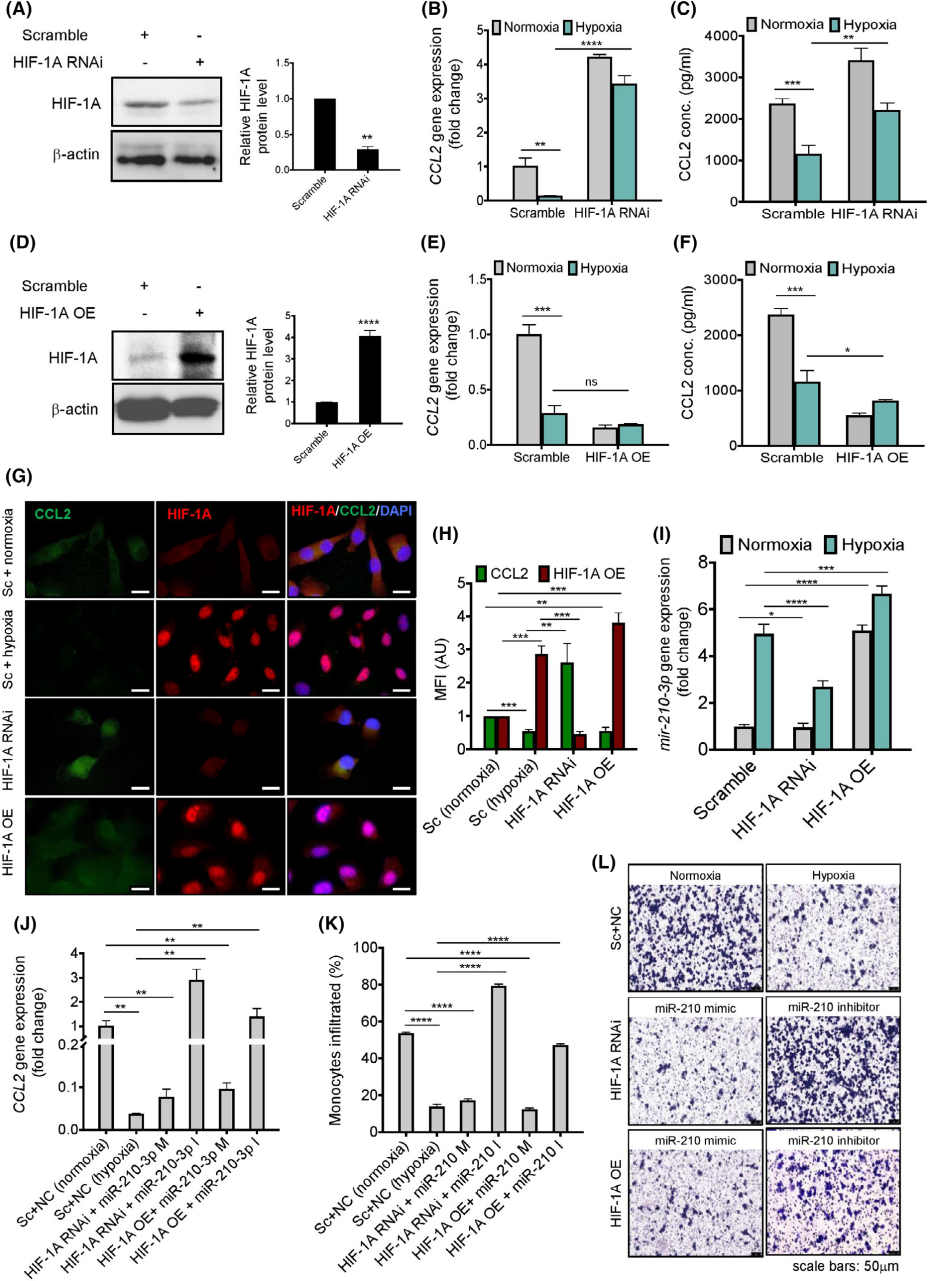
HIF‐1A regulates miR‐210‐3p mediated *CCL2* inhibition and monocyte infiltration in hypoxic A549 lung adenocarcinoma cells. (A) Immunoblots (left panel) with densitometric plots (right panel) showing the abundance of CCL2 and HIF‐1A proteins in A549 cells transfected with scramble or *HIF‐1A* RNAi plasmid. Data represent the mean ± SD of three independent experiments. Statistical significance was analyzed by Student's *t*‐test, **P < 0.01 vs Scramble. (B,C) RT‐qPCR analysis of *CCL2* gene expression (B), and ELISA showing CCL2 secretion into the culture medium (C) of A549 cells transfected with either scramble or *HIF‐1A* RNAi plasmid and incubated in 21% oxygen or 1% oxygen conditions for 24 h. Data represented as mean ± SD of three independent experiments. Statistical significance was analyzed by two‐way ANOVA, ****P < 0.0001, ***P < 0.001, **P < 0.01. (D) Immunoblots (left panel) with densitometric plots (right panel) showing the abundance of CCL2 and HIF‐1A proteins in A549 cells transfected with scramble plasmid and *HIF‐1A*‐ΟΕ (overexpression) plasmid. Data represented as mean ± SD of three independent experiments. Statistical significance was analyzed by Student's *t*‐test, ****P < 0.0001 vs Scramble. (E,F) RT‐qPCR analysis of relative *CCL2* gene expression (E), and ELISA showing CCL2 secretion into the culture medium (F) of A549 cells transfected with either scramble plasmid or *HIF‐1A*‐OE (overexpression) plasmid and incubated in 21% oxygen or 1% oxygen conditions for 24 h. Data represented as mean ± SD of three independent experiments. Statistical significance was analyzed by two‐way ANOVA, ***P < 0.001, *P < 0.05, ns, nonsignificant. (G,H) Representative immunofluorescence images (G) and their quantifications (H) showing CCL2 (green) and HIF‐1A (red) protein levels in A549 cells transfected with scramble or *HIF‐1A* RNAi or *HIF‐1A*‐OE plasmid and incubated under normoxic and hypoxic conditions for 24 h, respectively. Scale bars: 15 μm. Data represent the mean ± SD of three independent experiments. Statistical significance was analyzed by two‐way ANOVA, ***P < 0.001, **P < 0.01. (I) RT‐qPCR analysis showing *mir‐210‐3p* gene expression in A549 cells transfected with scramble or *HIF‐1A* RNAi or *HIF‐1A*‐OE plasmid and incubated under normoxic and hypoxic conditions for 24 h, respectively. Data represent mean ± SD of three independent experiments. Statistical significance was analyzed by two‐way ANOVA, ****P < 0.0001, ***P < 0.001, *P < 0.05. (J) RT‐qPCR analysis showing relative *CCL2* mRNA level in A549 cells transfected with scramble or *HIF‐1A* RNAi plasmid or *HIF‐1A*‐OE plasmid in the presence of miR‐210‐3p mimic (M) or miR‐210‐3p inhibitor (I) as indicated. Data represent mean ± SD of three independent experiments. Statistical significance was analyzed by one‐way ANOVA, **P < 0.01. (K,L) Representative images of THP‐1 monocytes migration (L) and their quantifications (K) from upper to lower surface of the transwell insert placed on the well containing A549 cells transfected with scramble or *HIF‐1A* RNAi or *HIF‐1A*‐OE plasmid in the presence of miR‐210‐3p mimic (M) or miR‐210‐3p inhibitor (I) as indicated. Scale bars: 50 μm. Data represent mean ± SD of three independent experiments. Statistical significance was analyzed by one‐way ANOVA, ****P < 0.0001.

To understand the regulation of *CCL2* expression in response to the HIF‐1A transcription factor, we performed *HIF‐1A* loss‐ and gain‐of‐function experiments using *HIF‐1A* RNAi and *HIF‐1A* overexpression plasmid, respectively. While *CCL2* mRNA and its protein expressions were significantly increased in *HIF‐1A* silenced A549 cells in a hypoxic state (Fig. 3A–C), forced expression of *HIF‐1A* notably downregulated the *CCL2* gene and protein expressions in normoxic condition (Fig. 3D–F). A similar trend was also witnessed in co‐immunofluorescence staining of HIF‐1A and CCL2 in *HIF‐1A* silenced and overexpressed A549 cells (Fig. 3G,H). These results indicate the role of HIF‐1A in *CCL2* expression. However, to determine whether the HIF‐1A‐induced downregulation of *CCL2* expression is mediated through miR‐210‐3p, we analyzed *mir‐210‐3p* expression in *HIF‐1A* silenced and overexpressed A549 lung cancer cells. A significant induction of *mir‐210‐3p* expression was observed in *HIF‐1A* overexpressed A549 cells, whereas substantial impairment was evident in *HIF‐1A* silenced cells (Fig. 3I), suggesting the possible involvement of miR‐210‐3p in HIF‐1A‐induced *CCL2* downregulation. To confirm this, *HIF‐1A* silenced and overexpressed A549 cells were transfected with miR‐210‐3p mimic or inhibitor, and we noticed high *CCL2* gene expression in the miR‐210‐3p inhibitor compared to the miR‐210‐3p mimic‐transfected *HIF‐1A* silenced cells (Fig. 3J). Conversely, a striking increase in *CCL2* expression was observed in the miR‐210‐3p inhibitor transfected *HIF‐1A* overexpressed cells compared to the miR‐210‐3p mimic transduced cells (Fig. 3J). It was also apparent from the transwell monocyte migration assay using the THP‐1 monocytes and A549 lung cancer cells. *HIF‐1A* silenced A549 cells when transfected with miR‐210‐3p mimic or miR‐210‐3p inhibitor and co‐cultured with THP‐1 monocytes, a significant decrease or increase of monocytes migration were observed, respectively (Fig. 3K,L). Delivery of miR‐210‐3p inhibitor to the *HIF‐1A* overexpressed A549 cells significantly restored the monocyte migration (Fig. 3K,L). All these results indicate the involvement of miR‐210‐3p in mediating HIF‐1A‐induced *CCL2* downregulation, and thus impairment of monocyte migration.

Since epigenetic regulations due to DNA methylation is considered one of the primary consequences of hypoxia in most tumors, we therefore also examined methylation‐dependent *CCL2* downregulation in response to hypoxic insult. DNA hypermethylation at promoters manifests an increase in 5‐methylcytosine (5‐mC) levels, causing transcriptional repression of targeted genes. Therefore, we initially analyzed the 5‐mC level in the lung cancer tissue section by immunofluorescence staining. An intense amount of 5‐mC was observed in cancerous tissue along with a higher level of HIF‐1A (Fig. S3A,B). We also quantified 5‐mC levels in the A549 cell line incubated with 21% oxygen (normoxia) and 1% oxygen (hypoxia). A significant increase in 5‐mC fluorescence was observed in hypoxic lung cancer cells (Fig. S3C,D). Concomitantly, DNMT activity was also increased in both the A549 cell line and 3D tumor spheroids in response to hypoxia (Fig. S3E,F). To analyze the gene expression of different DNMTs, we noticed an increasing trend in *DNMT‐1*, *DNMT‐3A*, and *DNMT‐3B* gene expression in hypoxic lung cancer cells compared to normoxia (Fig. S3G). These *DNMTs* gene expressions were decreased in *HIF‐1A* silenced cells and upregulated in *HIF‐1A* overexpressed A549 cancer cells (Fig. S3H–J), indicating the role of HIF‐1A in regulating *DNMTs* expression in a hypoxic state. Furthermore, we treated the hypoxic A549 cells with the 5‐Aza‐2′‐deoxycytidine, a DNMT inhibitor, to validate the role of DNA methylation in *CCL2* gene regulation in a hypoxic environment. Interestingly, we found that *CCL2* expression was significantly increased following 5‐Aza‐2′‐deoxycytidine treatment for 72 h in the presence of hypoxia (Fig. S3K,L). To understand the DNMT's role on *mir‐210‐3p* expression in hypoxic conditions, we found that 5‐azacytidine treatment did not significantly alter the *mir‐210‐3p* gene expression (Fig. S3M). All these results confirm that hypoxia‐induced HIF‐1A regulates *CCL2* expression through two independent axes of HIF‐1A–miR‐210‐3p–CCL2 and HIF‐1A–DNMT–CCL2 pathways. While comparing CCL2 protein levels in hypoxia treated miR‐210‐3p inhibitor transfected cells (Fig. 2M) with the cells incubated with 5‐Azacytidine in the presence of hypoxia (Fig. S3L), we noticed that hypoxia‐induced *CCL2* expression primarily governs through the HIF‐1A–miR‐210‐3p axis rather than the HIF‐1A–DNMT axis in A549 lung cancer cells.

### 3.4 Hypoxic tumor microenvironment of 3D lung adenocarcinoma spheroids regulates CCL2‐mediated monocyte infiltration and its TAM polarization

**Fig. 4:**
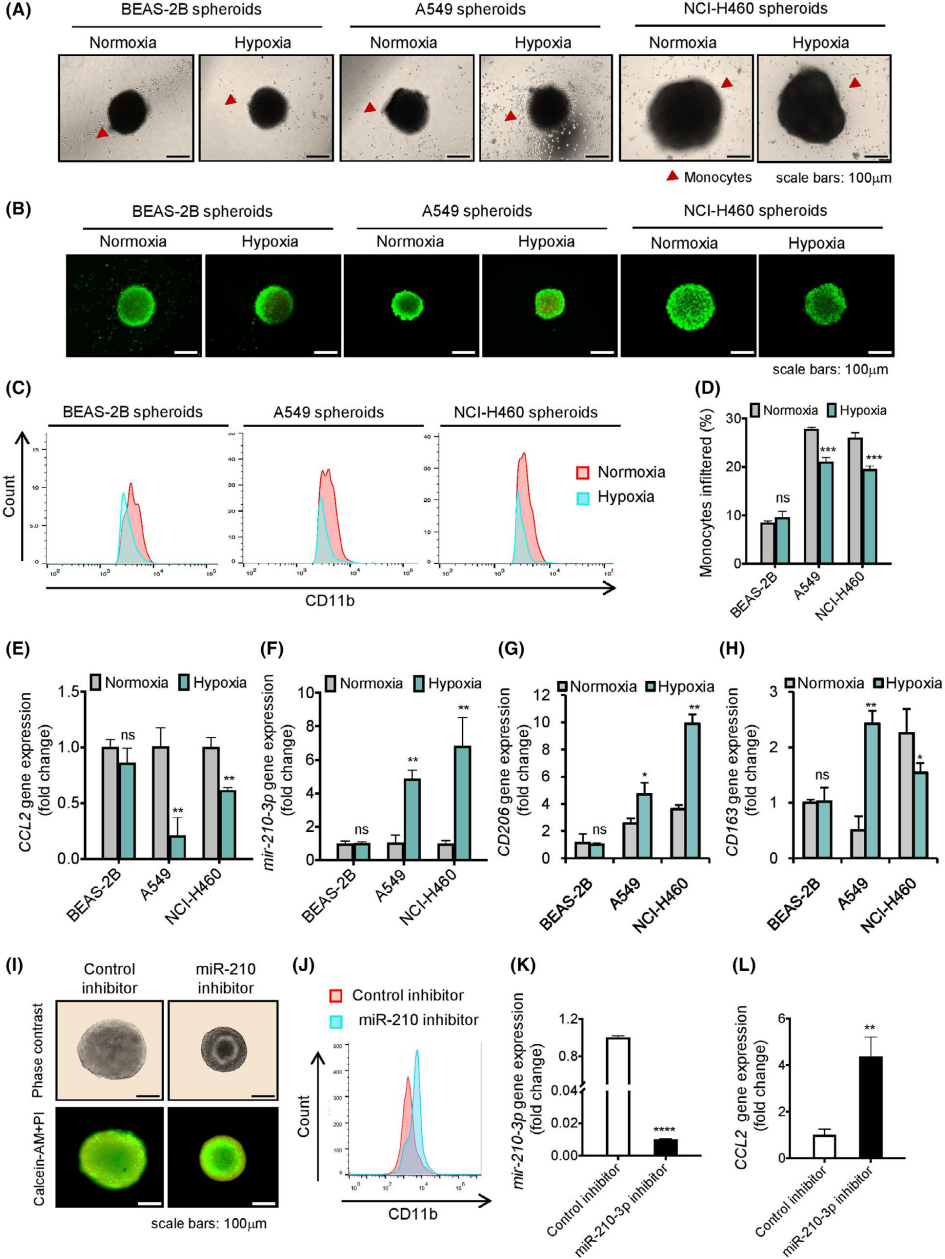
Hypoxic tumor microenvironment in 3D lung adenocarcinoma spheroids regulates CCL2 mediated monocyte infiltration and promotes TAM polarization (A,B) Representative phase‐contrast (A), and Calcein‐AM fluorescence staining (B) images of 3D spheroids of BEAS‐2B, A549, and NCI‐H460 cells on day 10 showing THP‐1 monocytes infiltrations in normoxic and hypoxic conditions for 24 h. Scale bars: 100 μm. (C,D) Flow cytometric analysis of CD11b level (C) and its quantifications (D) indicated THP‐1 monocytes infiltration in 3D spheroids of BEAS‐2B or A549 or NCI‐H460 cells incubated in normoxic and hypoxic conditions for 24 h. (E,F) RT‐qPCR analysis showing the relative abundance of *CCL2* (E), and *mir‐210‐3p* (F) gene expression in BEAS‐2B, A549, and NCI‐H460 cells spheroids under normoxic and hypoxic conditions. *GAPDH* and *U6 snRNA* were used as loading controls for *CCL2*, and *mir‐210‐3p* gene expression normalization, respectively. (G,H) RT‐qPCR analysis of *CD206* (G), and *CD163* (H) gene expression in BEAS‐2B, A549, and NCI‐H460 cell spheroids incubated with THP‐1 monocytes under normoxic and hypoxic conditions for 7 days. *GAPDH* was used as the loading control for normalization. (I) Representative phase‐contrast (upper panel) and Calcein‐AM fluorescence staining (lower panel) images of A549 cells spheroid transfected with control inhibitor or miR‐210‐3p inhibitor. Scale bars: 100 μm. (J) Flow cytometric analysis of CD11b level indicated THP‐1 monocytes infiltration in A549 cells spheroid transfected with control inhibitor or miR‐210‐3p inhibitor. (K,L) RT‐qPCR analysis of *mir‐210‐3p* (K) and *CCL2* (L) gene expression in A549 cells spheroid transfected with control inhibitor or miR‐210‐3p inhibitor. *GAPDH* and *U6 snRNA* were used as loading controls for *CCL2* and *mir‐210‐3p* gene expression normalization, respectively. Data represented as mean ± SD of three independent experiments. Statistical significance was analyzed by Student's *t*‐test, ****P < 0.0001, ***P < 0.001, **P < 0.01, *P < 0.05, ns, nonsignificant.

To understand the pathophysiological relationship between miR‐210‐3p and *CCL2* in the hypoxic tumor microenvironment, we established 3D spheroids of noncancerous lung epithelial cells BEAS‐2B or cancerous lung adenocarcinoma cells A549 and NCI‐H460 (Fig. 4A,B) and checked the rate of monocyte (THP‐1) infiltration in 21% oxygen (normoxic) and 1% oxygen (hypoxic) conditions at day 7. As shown in Fig. 4C,D, the percentage of monocytes infiltration was ~3‐fold more in A549 and NCI‐H460 spheroids than BEAS‐2B spheroid in normoxic conditions, but a significant reduction in monocyte infiltration appeared both in A549 and NCI‐H460 spheroids in response to 1% oxygen compared to 21% oxygen conditions. We further analyzed the *CCL2* and *mir‐210‐3p* gene expression in the 3D spheroids of BEAS‐2B, A549, and NCI‐H460 by quantitative PCR analysis. The *CCL2* gene expression was significantly downregulated in hypoxic A549 and NCI‐H460 spheroids as compared to normoxic ones (Fig. 4E), whereas the *mir‐210‐3p* gene expression was reversed (Fig. 4F). No significant alterations of *CCL2* and *mir‐210‐3p* expression were observed in BEAS‐2B spheroids either in normoxic and hypoxic conditions (Fig. 4E,F). Furthermore, we investigated the TAM polarization markers in infiltrated monocytes in 3D tumor spheroids. Direct contact with lung adenocarcinoma cells promotes monocytes phenotype skewing from M1 to the M2‐like/TAM state, as evidenced by the increased levels of *CD206* and *CD163* markers gene expression in 3D tumor spheroids of A549 and NCI‐H460 cells (Fig. 4G,H). Moreover, miR‐210‐3p inhibitor‐treated A549 tumor spheroids significantly altered the *mir‐210‐3p* and *CCL2* mRNA levels, reducing spheroid dimensions, and enhancing the monocyte population within the spheroids (Fig. 4I–L, Fig. S4A,B). Similarly, the HIF‐1A inhibitor (PX‐478)‐treated A549 tumor spheroids remarkably altered the *mir‐210‐3p* and *CCL2* gene expressions in hypoxic conditions (Fig. S4C,D). Moreover, miR‐210‐3p inhibitor‐treated tumor spheroids notably promote monocyte phenotypic skewing towards M1 polarized macrophages compared to control inhibitor transfected spheroids, as evident from the increased expression of *CD80*, *CD86*, *iNOS* and decreased expression of *CD206* and *CD163* (Fig. S4E–K). All these results demonstrate the role of miR‐210‐3p in the downregulation of *CCL2* expression in the hypoxic lung adenocarcinoma cells, which restricts monocyte migration and its TAM polarization in the hypoxic tumor microenvironment.

### 3.5 Inhibition of miR‐210‐3p promotes tumor regression in the A549 lung cancer xenograft zebrafish model

**Fig. 5:**
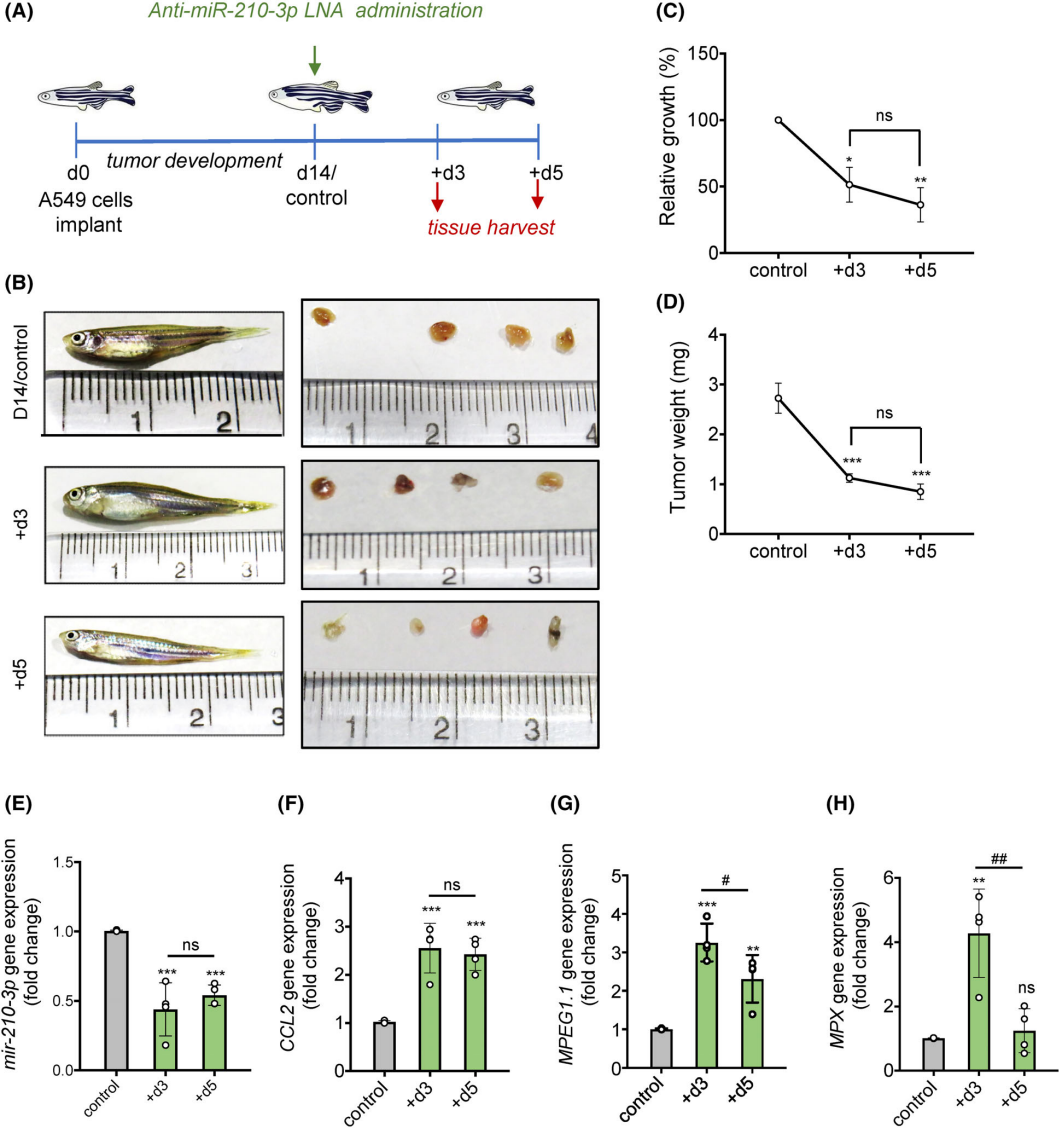
Anti‐miR‐210‐3p LNA delivery increases macrophage burden promoting tumor regression in the A549 lung cancer xenograft zebrafish model. (A) Schematic diagram representing experimental design indicating days of zebrafish A549 tumor xenograft model development followed by the administration of anti‐miR‐210‐3p LNA and collection of tumor tissue and blood samples from zebrafish. (B) Representative images of A549 tumor xenograft zebrafish (day 14/control) treated without or with anti‐miR‐210‐3p LNA for indicated timepoints (left panel) (*n* = 4) and the representative images of harvested tumors from these zebrafish (right panel). (C,D) Relative tumor growth (%) (C) and tumor weight (mg) (D) after treatment with anti‐miR‐210 LNA at indicated timepoints. (E,F) RT‐qPCR analysis of *mir‐210‐3p* (E) and *CCL2* (F) gene expression in tumor xenografts treated without or with anti‐miR‐210‐3p LNA and harvested at day 3 (+d3) and day 5 (+d5) post‐treatment. *U6 snRNA* and *β‐actin* were used as loading controls for *mir‐210‐3p* and *CCL2* gene expression normalization. (G,H) RT‐qPCR analysis showing relative abundance of *MPEG1.1* (G) and *MPX* (H) mRNA levels in tumor xenografts treated without or with anti‐miR‐210‐3p LNA for day 3 (+d3) and day 5 (+d5). *β‐actin* was used as a loading control for gene expression normalization. Data represented as mean ± SD. Statistical significance was analyzed by Student's *t*‐test, *P < 0.05, **P < 0.01, ***P < 0.001 vs control; ^#^ *P* < 0.05, ^##^ *P* < 0.01 vs +d3; ns, nonsignificant.

**Fig. 6:**
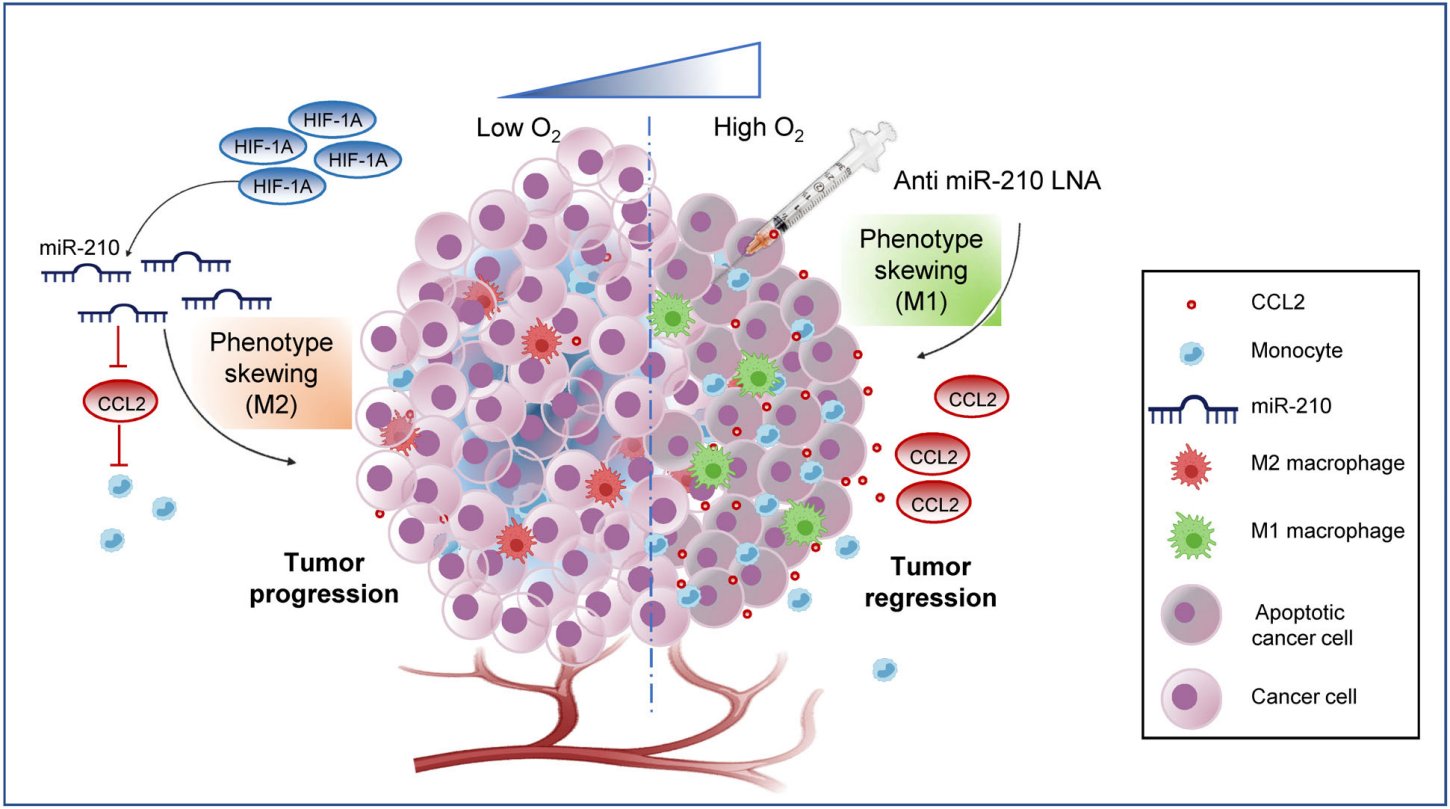
Model representing miR‐210‐3p as a potential therapeutic target for lung adenocarcinoma. Left panel: LUAD solid tumor exhibits hypoxia‐associated stabilization of HIF‐1A, which by promoting miR‐210‐3p level directly involved in downregulation of CCL2 and macrophage infiltration and also favors macrophage TAM phenotype supporting tumor progression. Right panel: Therapeutic intervention of anti‐miR210‐3p LNA augment CCL2 mediated monocyte infiltration and skewing towards macrophage antitumoral M1 phenotype leading to tumor regression.

To determine the therapeutic significance of miR‐210‐3p inhibition on lung adenocarcinoma (LUAD) tumorigenesis, we developed the LUAD tumor xenograft zebrafish model. We transplanted A549 cells in the peritoneum of immunocompromised adult zebrafish for 14 days and then delivered anti‐miR‐210‐3p LNA by intratumoral injection (Fig. 5A). Compared to the control group (day 14 tumor), we found that the delivery of anti‐miR‐210‐3p LNA significantly reduced tumor size, volume, and weight on day 3 and day 5 post‐treatment (Fig. 5B–D). We validated the *mir‐210‐3p* inhibition by examining its gene expression on day 3 and day 5 post‐treatment (Fig. 5E). Although *mir‐210‐3p* expression was significantly decreased on both day 3 and day 5 as compared to the control LNA treated group, however we did not notice any significant change on day 5 when compared with the day 3 group. This might be due to the limited half‐life of miRNA LNA. However, as we can see a marked reduction of *mir‐210‐3p* expression on day 3 compared to control, and this may lead to activating downstream regulatory pathways, which could be the reason for the tumor reduction on day 5. Moreover, we noticed a significant enhancement of *CCL2* gene expression in tumor samples on day 3 compared with control (Fig. 5F). We also measured the monocyte population inside the tumor after anti‐miR‐210‐3p LNA treatment. A significant increase of *MPEG1.1* and *MPX* gene expression in tumor xenograft on day 3 post‐treatment of anti‐miR‐210‐3p LNA indicated a higher monocyte population inside the tumor xenograft than control (Fig. 5G,H). The involvement of miR‐210‐3p in hypoxia‐induced tumor progression and the therapeutic application of anti‐miR‐210‐3p are presented in a schematic model diagram (Fig. 6). Thus, our results suggest the possible application of miR‐210‐3p inhibitor as a novel immunomodulatory therapeutic candidate against lung adenocarcinoma.

## 4 Discussion

Human solid tumors are invariably less oxygenated due to uncontrollable cell proliferation, altered metabolism, and abnormal blood vessels, leading to a hypoxic microenvironment [3, 4]. Tumor hypoxia induces stabilization of a transcription factor, hypoxia‐induced factor‐1A (HIF‐1A), that drives transcriptional responses in both stromal cells and cancer cells, thus promoting cancer progression [29]. Solid tumor development and progression are complex processes, which are not only induced by the accumulated genetic mutations in cancer cells but also regulated through the epigenetic changes influenced by the surrounding tumor microenvironment (TME) [30]. TME comprises innate and adaptive immune cells such as macrophages, dendritic cells, and T cells in solid tumors [31]. While adaptive immune cells can potentially inhibit tumor growth by eliminating cancer cells, the innate immune cells also promote the antitumor activity as infiltrating monocytes involved in tumor regression [32]. HIFs are tightly connected with the inflammatory responses, and hypoxia can directly or indirectly regulate the functions of almost all immune cell types, thereby influencing tumor development [33, 34]. Therefore, targeting tumor macrophages is an attractive strategy for solid tumor therapeutic intervention [35]. This study found that hypoxia‐induced miR‐210‐3p can limit the monocyte infiltration in lung tumors by modulating *CCL2* expression, thus potentiating tumor progression in LUAD.

In this study we created an *in vitro* hypoxia model by exposing the cells to a 1% oxygen level and compared it with the cells exposed to a 21% oxygen level (universally considered “Normoxia”) and used in the present investigation. However, it could be noted here that the oxygen level drops in arterial blood, and by the time it reaches peripheral tissues, the median oxygen level ranges from 3.4% to 6.8%, and therefore the 5.6% oxygen level represented as “Physoxia” (i.e., the oxygen level in normal peripheral tissues) [3]. CCL2 is a potent chemoattractant for monocytes that leads to their infiltration into the tumors [9]. Compelling evidence demonstrated both protumoral and antitumoral effects of CCL2, while CCL2 exhibits tumor‐promoting activities by the recruitment of TAMs and myeloid‐derived suppressor cells (MDSCs), as well as the stimulation of tumor cell invasiveness; the antitumoral potential of CCL2 majorly contributed due to its ability of immune stimulation and inflammation against the tumor [36]. *CCL2* expression can be modulated by the transcriptional regulation of HIF‐1A and works via the CCR2 axis on the target cells [17, 37]. HIF‐1A and CCL2 have been shown to play a pivotal role in the recruitment of monocytes and cancer progression [38]; however, the interrelationship between these two factors varies in solid tumor types [12]. Our study has revealed the role of HIF‐1A on *CCL2* expression when examining the *HIF‐1A* silenced or overexpressed lung adenocarcinoma cell lines. *CCL2* expression was significantly enhanced in *HIF‐1A* silenced cells, whereas forced expression of *HIF‐1A* strikingly decreased the expression of *CCL2* in A549 cells, suggesting the involvement of HIF‐1A in the regulation of *CCL2* gene expression. This result corroborated the previous observation in astrocytes reported by Petrovic et al., 2007 [17]. Moreover, HIF‐1A and/or HIF‐2A expression levels are frequently elevated and associated with the poor prognosis of human malignancies [39] and, on the contrary, HIF‐3A has been classified as a dominant‐negative regulator of HIF‐1A/HIF‐2A [40].

In our study we found that HIF‐1A‐induced downregulation of *CCL‐2* expression is mediated through miR‐210‐3p, a well‐known hypoxamiR [20, 24]. A significant induction or impairment of *mir‐210‐3p* expression was observed in *HIF‐1A* overexpressed or silenced A549 cells, respectively, suggesting that miR‐210‐3p might play a critical role in HIF‐1A‐induced *CCL2* downregulation. Analyzing the miR target reporter luciferase assay using the miR‐210‐3p inhibitor and target protector revealed that miR‐210‐3p directly binds to the *CCL2*‐3′UTR and silences its expression. However, such an attribute was significantly abrogated by the mutation at the *CCL2*‐3′UTR seed region, indicating a direct involvement of hypoxamiR‐210‐3p on *CCL2* expression and monocyte migration.

For further confirmation, we transfected miR‐210‐3p mimic in *HIF‐1A* silenced lung cancer cells, and *HIF‐1A* overexpressed lung cancer cells were transfected with the miR‐210‐3p inhibitor. We noticed a significant inhibition of *CCL2* expression and monocyte migration in the miR‐210‐3p mimic transfected *HIF‐1A* silenced cells; whereas, the miR‐210‐3p inhibitor transfected *HIF‐1A* overexpressed cells significantly restored the *CCL2* expression and monocyte migration. However, it is intriguing to note that the miR‐210‐3p inhibitor did not completely rescue the CCL2 expression and monocyte migration in *HIF‐1A* overexpressed A549 cells, suggesting a possible involvement of some other factors that may be associated with HIF‐1A‐mediated *CCL2* downregulation.

Recent evidence suggests that hypoxia dynamically regulates epigenetic modification to drive the malignant behavior of cancer cells [41, 42]. Our study found a striking induction of the 5‐mC level in adenocarcinoma patient samples and in hypoxic A549 cells, along with the increment of DNMT activity in hypoxic tumor spheroids, indicating the involvement of DNA methylation. To establish that *CCL2* expression is under the influence of its promoter methylation in the hypoxic lung tumor microenvironment, we used the DNMT inhibitor 5‐Aza‐2′‐deoxycytidine in the study and found an increase in the CCL2 level, establishing possible involvement of the *CCL2* promoter methylation in hypoxia‐induced *CCL2* downregulation.

The tumor microenvironment is vastly different from the conditions used in traditional 2D monolayer‐based cancer cell culture that lacks a natural tumor milieu [43]. The use of 3D tumor spheroids will provide a more accurate and closer representation of human solid tumor architecture and biology, such as cell–cell communication and hypoxia, respectively, and therefore it represents a more appropriate model for preclinical studies [44]. We thus used noncancerous (BEAS‐2B) and cancerous lung adenocarcinoma cell lines (A549 and NCI‐H460) to generate respective 3D spheroids and analyzed the monocyte infiltration in normoxic and hypoxic conditions. Interestingly, a reduction of monocyte migration is associated with the increased level of M2 polarization markers such as CD206 and CD163 in the hypoxic condition, suggesting that the hypoxic tumor microenvironment facilitates the skewing of monocyte polarization, inhibiting its tumoricidal activity. Also, a marked reduction of *CCL2* expression along with the induction of *mir‐210‐3p* gene expression was observed in hypoxic tumor spheroids as compared to the normoxic state. Application of the HIF‐1A blocker or miR‐210‐3p inhibitor showed a notable suppression of tumor cell growth in 3D spheroids along with the induction of *CCL2* expression, monocyte migration, and switching macrophage polarity. These results replicated our findings in monolayer NSCLC culture experiments, thus showing the potential of miR‐210‐3p and HIF‐1A inhibitors in preventing hypoxia‐induced inhibition of monocyte infiltration in the lung tumor microenvironment and the growth of lung cancer spheroids.

In many solid tumor types, tumor‐associated macrophages (TAMs) are the most abundant population of tumor‐infiltrating immune cells in TME [45]. Moreover, the TAMs population is strongly associated with poor survival in solid tumor patients [46]. TAMs play significant roles in tumor initiation, growth, development, and metastasis by secreting a wide variety of cytokines, growth factors, inflammatory substrates, and proteolytic enzymes [46, 47]. The number of infiltrating monocytes significantly dropped in hypoxic LUAD solid tumors; thus, it might be one of the reasons why immunotherapy failed to fully function in this type of tumor. We therefore aimed to increase the number of infiltrating monocytes by reducing the expression of monocyte chemoattractant CCL2 by therapeutic application of the miR‐210‐3p inhibitor in the LUAD xenograft zebrafish model. Over the last decade, the adult zebrafish xenograft model gained massive popularity in the scientific community as an alternative *in vivo* animal model in cancer research [48]. It was evident that zebrafish orthologs for ~80% of genes is related to human disease with significant conservation of different molecular pathways in zebrafish and humans. To date, several different types of zebrafish cancer xenografts were established for their use, not only as an alternative to mouse models, but also for their great promise in functional studies of small molecules and personalized medicine [49, 50]. Our study evaluated the therapeutic efficacy of the miR‐210‐3p inhibitor on tumor growth and monocyte migration in the zebrafish LUAD xenograft model. After intratumoral administration of anti‐miR‐210‐3p LNA in zebrafish A549 xenografts, the tumor size was reduced significantly with an increased population of monocytes in these xenografts. This suggests that increased infiltrating monocytes might play a tumoricidal role against LUAD solid tumors. This study opens the opportunity to alter the immune regulation of the lung tumor microenvironment by applying a specific miR inhibitor, and thus is potentially beneficial for lung cancer management. Considering the higher plasticity and flexibility of monocytes, alternative reprogramming of tumor‐resident TAM phenotype from its existing protumoral phenotype to a tumoricidal M1 phenotype could also be a viable option for lung cancer management; therefore, further study is required to target this approach.

## 5 Conclusions

In conclusion, the present study provides evidence that hypoxia‐associated induction of HIF‐1A markedly enhanced miR‐210‐3p expression, which directly governs CCL2 downregulation in lung adenocarcinoma resulting in the impairment of monocyte infiltration and the stimulation of macrophage TAM polarization. Moreover, it also revealed that the application of the miR‐210‐3p inhibitor could serve as a novel immunomodulatory therapeutic agent for treating lung adenocarcinoma patients and it may also be helpful to treat other carcinoma solid tumors.

## Conflict of interest

The authors declare no conflict of interest.

## Author contributions

DP and LA are involved in study conception and design. LA, DeP, SR, SN, AKV, and NS are involved in methodology and acquisition of data. LA, DeP, SR, SN, SD, NS, AC, AKV, and DP are involved in analysis and interpretation of data. NS is involved in collection of patients' samples and clinical parameters. LA, SD, NS, AC, and DP are involved in writing and critical revision of the article. DP is involved in study supervision. All authors read and approved the final article.

### Peer review

The peer review history for this article is available at https://publons.com/publon/10.1002/1878-0261.13260.

## Supporting information


**Fig. S1.** Hypoxia‐induced HIF‐1A and miR‐210‐3p regulate *CCL2* expression in lung adenocarcinoma. (A) Heat map indicating *HIF‐1A* and *CCL2* mRNA expression z‐scores in 510 lung adenocarcinoma (TCGA, PanCancer atlas) patient samples and stratification of tumor samples in normoxic, hypoxic, and intermediate categories based on unsupervised hierarchical clustering (Ward.D of the clusth function in R's stats package) on normalized RSEM values for *HIF‐1A* gene that make up the hypoxia metagene signature. (B) Inverse correlation between *HIF‐1A* and *CCL2* RSEM values in 55 hypoxic patients stratified from TCGA data (p = 0.0001, Pearson r = −0.7283, r^2^ = 0.5304). (C) Comparison of *HIF‐1A* and *CCL2* RSEM values indicating low *CCL2* expression in hypoxic tumors. (D) Representative images of immunofluorescent staining of noncancerous (n = 3) and cancerous lung adenocarcinoma (n = 3) tissue sections for visualization of VE‐cadherin (green) and HIF‐1A (red). Nuclei were counterstained with DAPI (blue). N = 3 images were taken per sample. (E) The mean fluorescence intensity (MFI) of VE‐cadherin and HIF‐1A in noncancerous and lung adenocarcinoma tissue sections were quantified as relative values of 3 patients (n = 3) and 3 values per sample (N = 3) using Image J program and represented as a bar diagram. (F) Immunoblot and ponceau staining showing the abundance of CCL2 protein in pleural fluid samples (60 μg protein) from noncancerous and cancerous lung adenocarcinoma patients.

**Fig. S2.** Hypoxia stimulates*CCL2* expression in lung adenocarcinoma cell lines. (A) Immunoblots indicating the abundance of CCL2 and HIF‐1A proteins in A549 and NCI‐H460 cell lines incubated with indicated levels of oxygen for 24 h. β‐actin was used as a loading control. (B,C) RT‐qPCR analysis showing the relative abundance of *CCL2* mRNA level in A549 cells treated either with indicated concentrations of CoCl_2_ for 24 h (B) or exposed to 1% oxygen for indicated time points (C). *GAPDH* was used as a loading control for normalization. (D‐F) RT‐qPCR analysis showing the relative abundance of *CCL2* mRNA expression in A549 and NCI‐H460 cells (D), ELISA showing CCL2 secretion into the culture medium of A549 and NCI‐H460 cells (E), and immunoblots showing the abundance of CCL2 and HIF‐1A proteins in A549 cells (F) incubated in the presence of 21% oxygen or 1% oxygen conditions. *GAPDH* and β‐actin served as loading controls for RT‐qPCR and immunoblotting, respectively. (G) Determination of A549 and NCI‐H460 cell viability in response to indicated concentrations of CoCl2 by MTT assay. (H) Interaction between miR‐210‐3p and *CCL2* mRNA was presented in graphic format and minimum free energy (mfe) was calculated using RNAhybrid online tool. (I) Representation of wildtype CCL2‐3’UTR seed sequence (in bold) and its binding site on miR‐210‐3p along with the mutated CCL2‐3’UTR seed sequence (in red) and the sequence of 60 nucleotides long target protector used in the present investigation. (J) miR target reporter luciferase assay showing relative CCL2‐3’UTR luciferase activity in control or miR‐210‐3p mimic transfected A549 cells treated without or with 60 nucleotides long target protector. Data represented as mean ± S.D. of three independent experiments. **p < 0.01, ***p < 0.001, and ****p < 0.0001.

**Fig. S3.** HIF‐1A‐induced DNMT activation regulates *CCL2* expression in hypoxic lung adenocarcinoma. (A,B) Representative H&E staining (left panel) and immunofluorescence staining (right panel) images showing HIF‐1A (red) and 5‐mC (green) levels (A) and their quantifications (B) in noncancerous (n = 3), and lung adenocarcinoma tissue samples (n = 3). Nuclei were counterstained with DAPI (blue). N = 3 images were taken per sample. Scale bars: 75 μm. (C,D) Representative immunofluorescence staining images showing HIF‐1A (red) and 5‐mC (green) levels (C), and their quantifications (D) in A549 cells incubated in normoxic or hypoxic conditions for 24 h. Scale bars: 15 μm. (E) Timedependent DNMT (OD/h/mg) activity in A549 cells in response to hypoxic conditions for indicated time periods. (F) DNMT activity (OD/h/mg) was measured in A549 tumor spheroids exposed to normoxic or hypoxic conditions for24 h. (G) RT‐qPCR analysis of *DNMT‐1*, *DNMT‐3A*, and *DNMT‐3B* gene expression in A549 tumor spheroids incubated in normoxic or hypoxic conditions for 24 h. *GAPDH* was used as a loading control for normalization. (H‐J) RT‐qPCR analysis of *DNMT‐1* (H), *DNMT‐3A* (I), and *DNMT‐3B* (J) gene expression in A549 cells transfected with scramble or HIF‐1A RNAi or HIF‐1A OE plasmid and exposed to normoxic or hypoxic conditions for 24 h. *GAPDH* was used as a loading control for normalization. (K) RT‐qPCR analysis of *CCL2* mRNA levels in A549 cells treated without or with DNA methylation inhibitor 5‐Azacytidine for 72 h followed by the incubations in normoxic or hypoxic conditions for 24 h. *GAPDH* was used as a loading control for normalization. (L) ELISA showing CCL2 secretion into the culture medium of A549 cells treated with PBS (vehicle control) and with DNA methylation inhibitor 5‐Azacytidine for 72 h followed by the incubations in normoxic or hypoxic conditions for 24 h. (M) RT‐qPCR analysis of *mir‐210‐3p* gene expression in A549 cells treated with DNA methylation inhibitor 5‐Azacytidine for 72 h followed by the incubations in normoxic or hypoxic conditions for 24 h. *U6 snRNA* was used as an internal reference control. Data represent the mean ± S.D. of three independent experiments. *p < 0.05, **p < 0.01, ***p < 0.001, and ****p < 0.0001.

**Fig. S4.** Regulation of *CCL2* and *mir‐210‐3p* expression in response to HIF inhibitor and the effect of the miR‐210‐3p inhibitor on macrophage M1 phenotype switching (A) Bar diagram representing diameter (μm) of A549 tumor spheroids when treated with control inhibitor or miR‐210‐3p inhibitor. (B) Percentage of THP‐1 monocytes infiltration in 3D spheroids treated with control and miR‐210‐3p inhibitor for 48 h. (C,D) RT‐qPCR analysis of *CCL2* (C), and *mir‐210‐3p* (D) gene expression in A549 tumor spheroids treated without or with HIF‐1A inhibitor PX478 for 24 h. (E,F) Flow cytometric analysis of CD80 and CD206 levels (E) and their quantifications (F) in THP‐1 infiltrated (7 days) tumor spheroids transfected with control inhibitor or miR‐210‐3p inhibitor. (G‐K) RT‐qPCR analysis of *CD80* (G), *CD86* (H), *iNOS* (I), *CD206* (J) and *CD163* (K) gene expression in THP‐1 infiltrated (7 days) A549 tumor spheroids transfected with control inhibitor or miR‐210‐3p inhibitor. *GAPDH* and *U6 snRNA* were used as loading controls for all mRNAs and *mir‐210‐3p* gene expression normalization, respectively. Data represent mean ± S.D. of three independent experiments. *p < 0.05, **p < 0.01, ***p < 0.001, and ****p < 0.0001.

**Table S1.** Clinical characteristics of the lung adenocarcinoma and noncancerous patients.
**Table S2.** List of primers with sequence details.

Supplementary material

## Data Availability

The data that support the findings of this study are available from the corresponding author (durba.pal@iitrpr.ac.in) upon reasonable request.
