# Supplementary figures and images for: Hypoxia‐induced miR‐210‐3p expression in lung adenocarcinoma potentiates tumor development by regulating CCL2 mediated monocyte infiltration
Source: Mol Oncol. 2024 Mar 22;18(5):1278–300. doi: 10.1002/1878-0261.13260 (PMC11077004; doi:10.1002/1878-0261.13260)

Figure S1

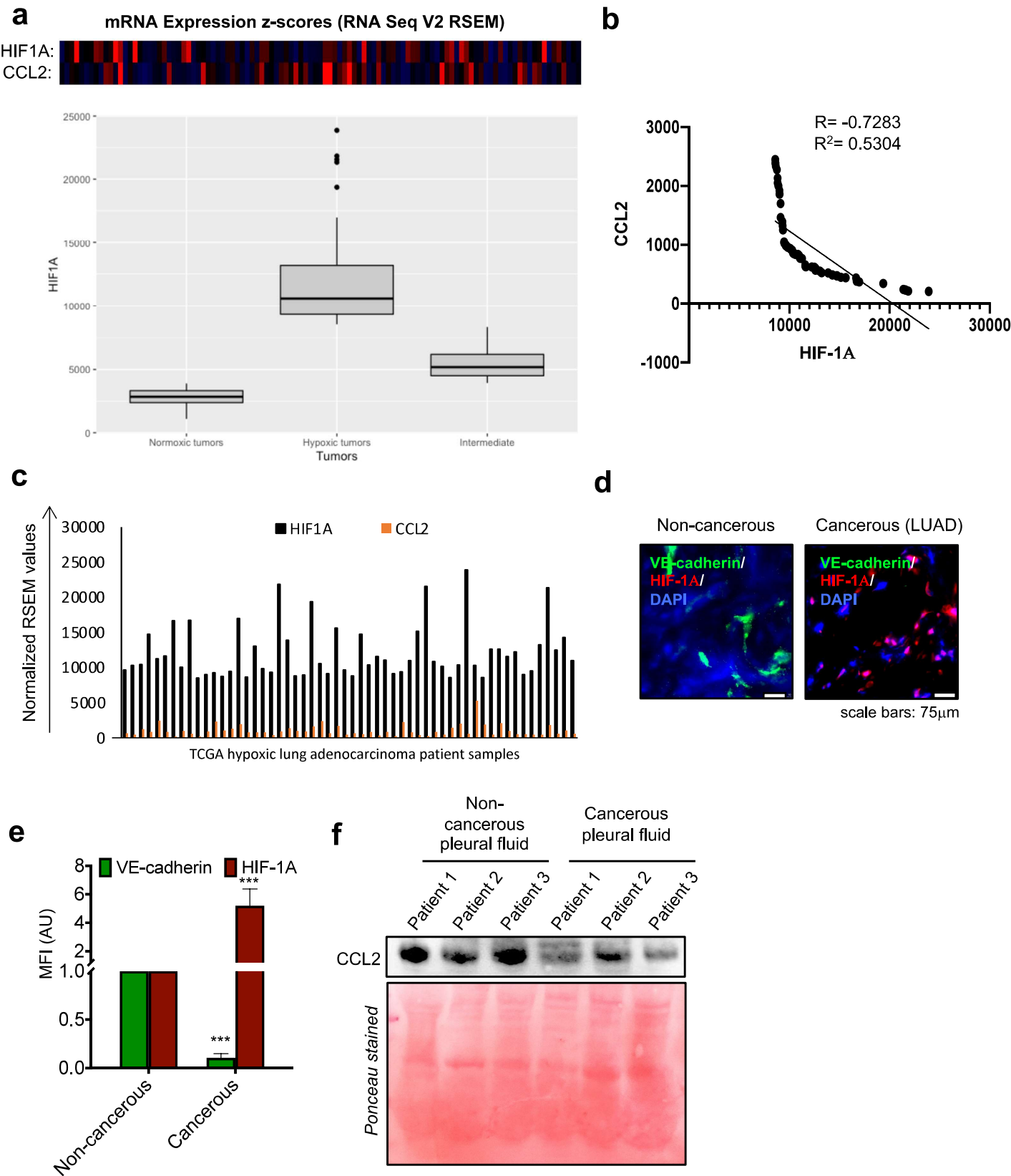

Supplement: Supplementary file 1 — Fig. S1. Hypoxia‐induced HIF‐1A and miR‐210‐3p regulate CCL2 expression in lung adenocarcinoma. (A) Heat map indicating HIF‐1A and CCL2 mRNA expression z‐scores in 510 lung adenocarcinoma (TCGA, PanCancer atlas) patient samples and stratification of tumor samples in normoxic, hypoxic, and intermediate categories based on unsupervised hierarchical clustering (Ward.D of the clusth function in R's stats package) on normalized RSEM values for HIF‐1A gene that make up the hypoxia metagene signature. (B) Inverse correlation between HIF‐1A and CCL2 RSEM values in 55 hypoxic patients stratified from TCGA data (p = 0.0001, Pearson r = −0.7283, r2 = 0.5304). (C) Comparison of HIF‐1A and CCL2 RSEM values indicating low CCL2 expression in hypoxic tumors. (D) Representative images of immunofluorescent staining of noncancerous (n = 3) and cancerous lung adenocarcinoma (n = 3) tissue sections for visualization of VE‐cadherin (green) and HIF‐1A (red). Nuclei were counterstained with DAPI (blue). N = 3 images were taken per sample. (E) The mean fluorescence intensity (MFI) of VE‐cadherin and HIF‐1A in noncancerous and lung adenocarcinoma tissue sections were quantified as relative values of 3 patients (n = 3) and 3 values per sample (N = 3) using Image J program and represented as a bar diagram. (F) Immunoblot and ponceau staining showing the abundance of CCL2 protein in pleural fluid samples (60 μg protein) from noncancerous and cancerous lung adenocarcinoma patients. [file MOL2-18-1278-s006.pdf]

Figure S2

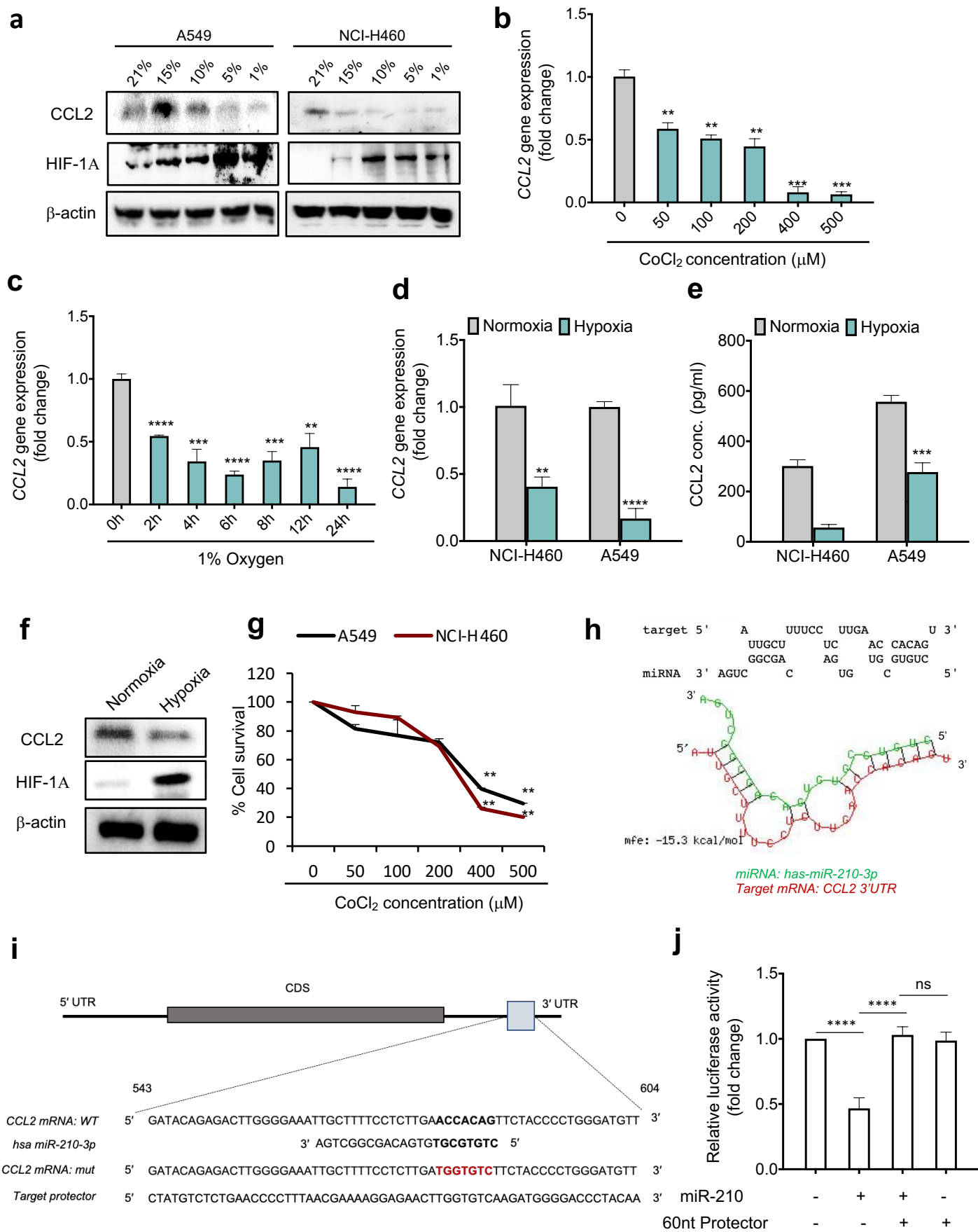

Supplement: Supplementary file 2 — Fig. S2. Hypoxia stimulatesCCL2 expression in lung adenocarcinoma cell lines. (A) Immunoblots indicating the abundance of CCL2 and HIF‐1A proteins in A549 and NCI‐H460 cell lines incubated with indicated levels of oxygen for 24 h. β‐actin was used as a loading control. (B,C) RT‐qPCR analysis showing the relative abundance of CCL2 mRNA level in A549 cells treated either with indicated concentrations of CoCl2 for 24 h (B) or exposed to 1% oxygen for indicated time points (C). GAPDH was used as a loading control for normalization. (D‐F) RT‐qPCR analysis showing the relative abundance of CCL2 mRNA expression in A549 and NCI‐H460 cells (D), ELISA showing CCL2 secretion into the culture medium of A549 and NCI‐H460 cells (E), and immunoblots showing the abundance of CCL2 and HIF‐1A proteins in A549 cells (F) incubated in the presence of 21% oxygen or 1% oxygen conditions. GAPDH and β‐actin served as loading controls for RT‐qPCR and immunoblotting, respectively. (G) Determination of A549 and NCI‐H460 cell viability in response to indicated concentrations of CoCl2 by MTT assay. (H) Interaction between miR‐210‐3p and CCL2 mRNA was presented in graphic format and minimum free energy (mfe) was calculated using RNAhybrid online tool. (I) Representation of wildtype CCL2‐3’UTR seed sequence (in bold) and its binding site on miR‐210‐3p along with the mutated CCL2‐3’UTR seed sequence (in red) and the sequence of 60 nucleotides long target protector used in the present investigation. (J) miR target reporter luciferase assay showing relative CCL2‐3’UTR luciferase activity in control or miR‐210‐3p mimic transfected A549 cells treated without or with 60 nucleotides long target protector. Data represented as mean ± S.D. of three independent experiments. **p < 0.01, ***p < 0.001, and ****p < 0.0001. [file MOL2-18-1278-s002.pdf]

**Figure S3**

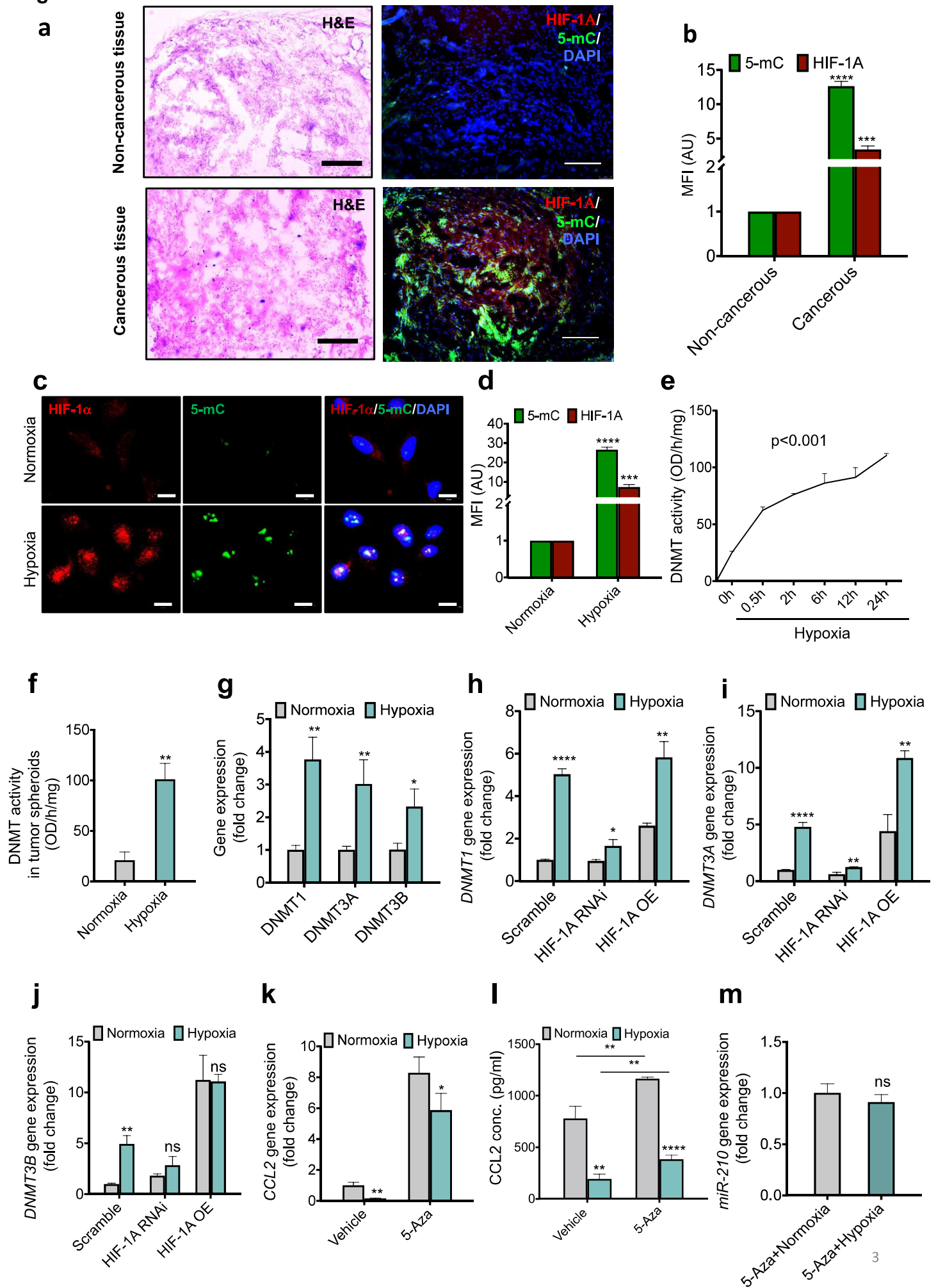

Supplement: Supplementary file 3 — Fig. S3. HIF‐1A‐induced DNMT activation regulates CCL2 expression in hypoxic lung adenocarcinoma. (A,B) Representative H&E staining (left panel) and immunofluorescence staining (right panel) images showing HIF‐1A (red) and 5‐mC (green) levels (A) and their quantifications (B) in noncancerous (n = 3), and lung adenocarcinoma tissue samples (n = 3). Nuclei were counterstained with DAPI (blue). N = 3 images were taken per sample. Scale bars: 75 μm. (C,D) Representative immunofluorescence staining images showing HIF‐1A (red) and 5‐mC (green) levels (C), and their quantifications (D) in A549 cells incubated in normoxic or hypoxic conditions for 24 h. Scale bars: 15 μm. (E) Timedependent DNMT (OD/h/mg) activity in A549 cells in response to hypoxic conditions for indicated time periods. (F) DNMT activity (OD/h/mg) was measured in A549 tumor spheroids exposed to normoxic or hypoxic conditions for24 h. (G) RT‐qPCR analysis of DNMT‐1, DNMT‐3A, and DNMT‐3B gene expression in A549 tumor spheroids incubated in normoxic or hypoxic conditions for 24 h. GAPDH was used as a loading control for normalization. (H‐J) RT‐qPCR analysis of DNMT‐1 (H), DNMT‐3A (I), and DNMT‐3B (J) gene expression in A549 cells transfected with scramble or HIF‐1A RNAi or HIF‐1A OE plasmid and exposed to normoxic or hypoxic conditions for 24 h. GAPDH was used as a loading control for normalization. (K) RT‐qPCR analysis of CCL2 mRNA levels in A549 cells treated without or with DNA methylation inhibitor 5‐Azacytidine for 72 h followed by the incubations in normoxic or hypoxic conditions for 24 h. GAPDH was used as a loading control for normalization. (L) ELISA showing CCL2 secretion into the culture medium of A549 cells treated with PBS (vehicle control) and with DNA methylation inhibitor 5‐Azacytidine for 72 h followed by the incubations in normoxic or hypoxic conditions for 24 h. (M) RT‐qPCR analysis of mir‐210‐3p gene expression in A549 cells treated with DNA methylation inhibitor 5‐Azacytidine for 72 h fol [file MOL2-18-1278-s003.pdf]

Figure S4

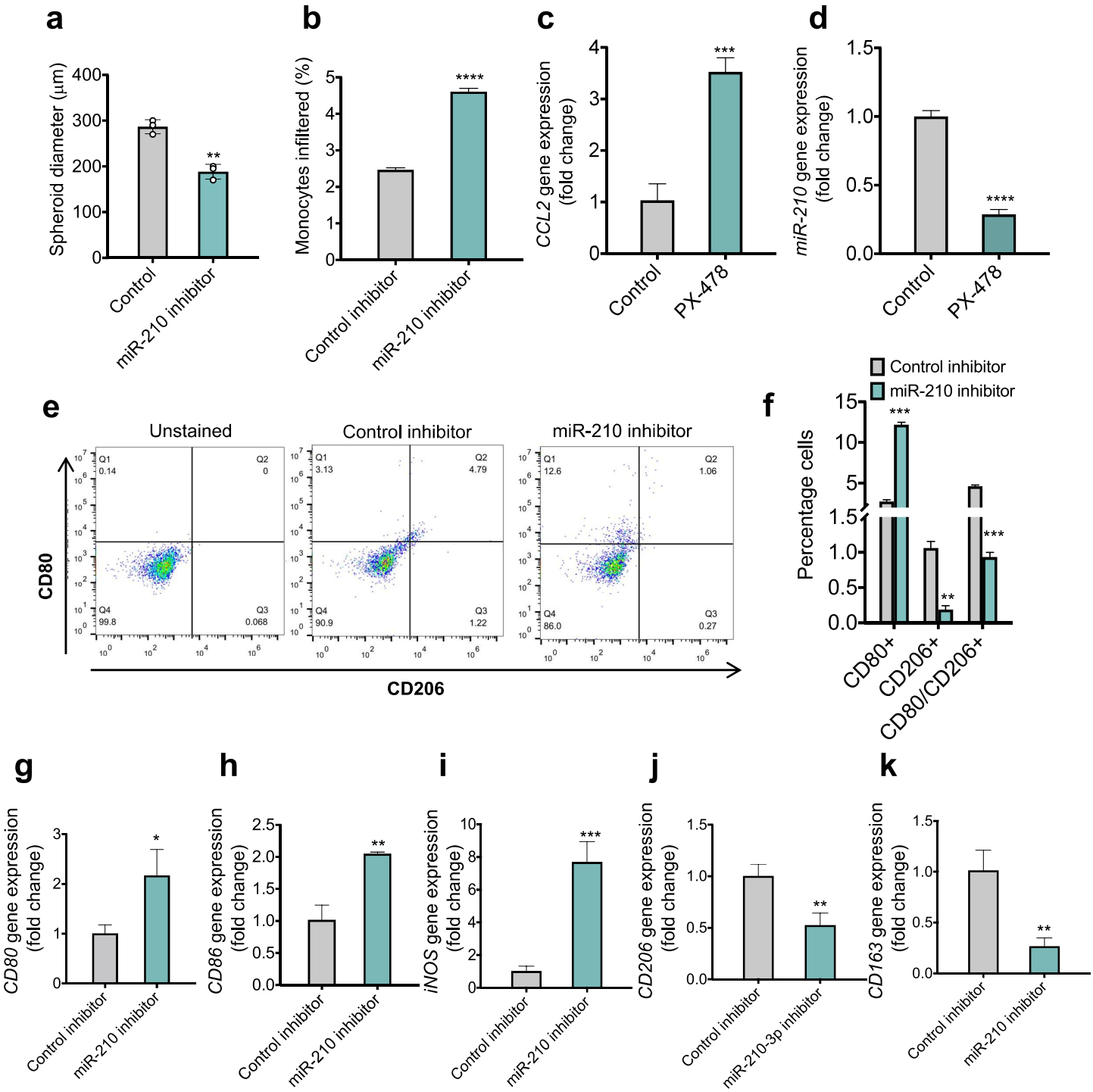

Supplement: Supplementary file 4 — Fig. S4. Regulation of CCL2 and mir‐210‐3p expression in response to HIF inhibitor and the effect of the miR‐210‐3p inhibitor on macrophage M1 phenotype switching (A) Bar diagram representing diameter (μm) of A549 tumor spheroids when treated with control inhibitor or miR‐210‐3p inhibitor. (B) Percentage of THP‐1 monocytes infiltration in 3D spheroids treated with control and miR‐210‐3p inhibitor for 48 h. (C,D) RT‐qPCR analysis of CCL2 (C), and mir‐210‐3p (D) gene expression in A549 tumor spheroids treated without or with HIF‐1A inhibitor PX478 for 24 h. (E,F) Flow cytometric analysis of CD80 and CD206 levels (E) and their quantifications (F) in THP‐1 infiltrated (7 days) tumor spheroids transfected with control inhibitor or miR‐210‐3p inhibitor. (G‐K) RT‐qPCR analysis of CD80 (G), CD86 (H), iNOS (I), CD206 (J) and CD163 (K) gene expression in THP‐1 infiltrated (7 days) A549 tumor spheroids transfected with control inhibitor or miR‐210‐3p inhibitor. GAPDH and U6 snRNA were used as loading controls for all mRNAs and mir‐210‐3p gene expression normalization, respectively. Data represent mean ± S.D. of three independent experiments. *p < 0.05, **p < 0.01, ***p < 0.001, and ****p < 0.0001. [file MOL2-18-1278-s001.pdf]

# Supporting document

## IgG loading control

Related to Fig. 1A

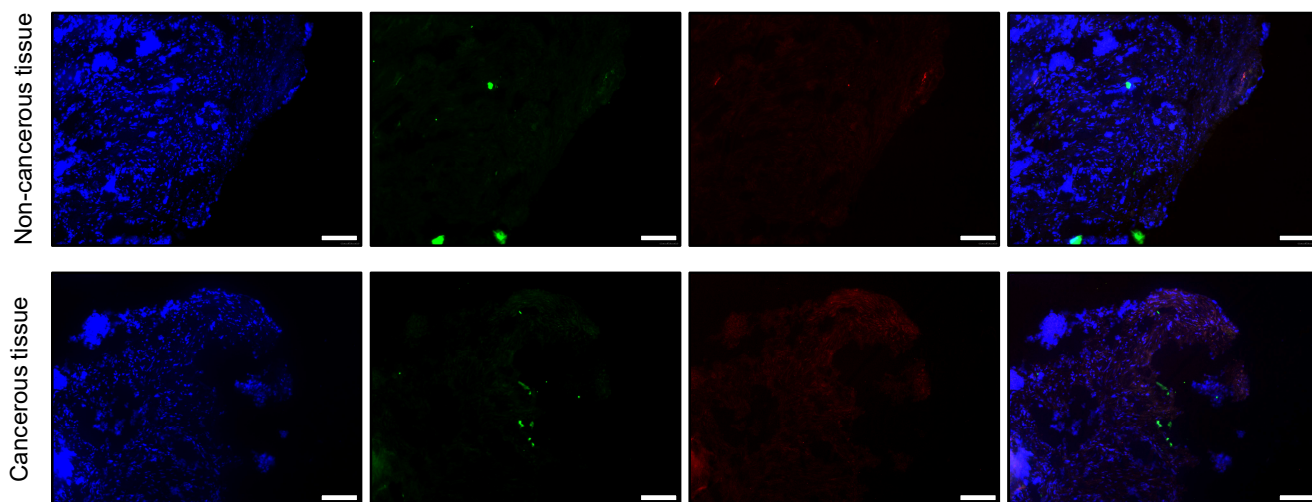

Related to Fig. S3A

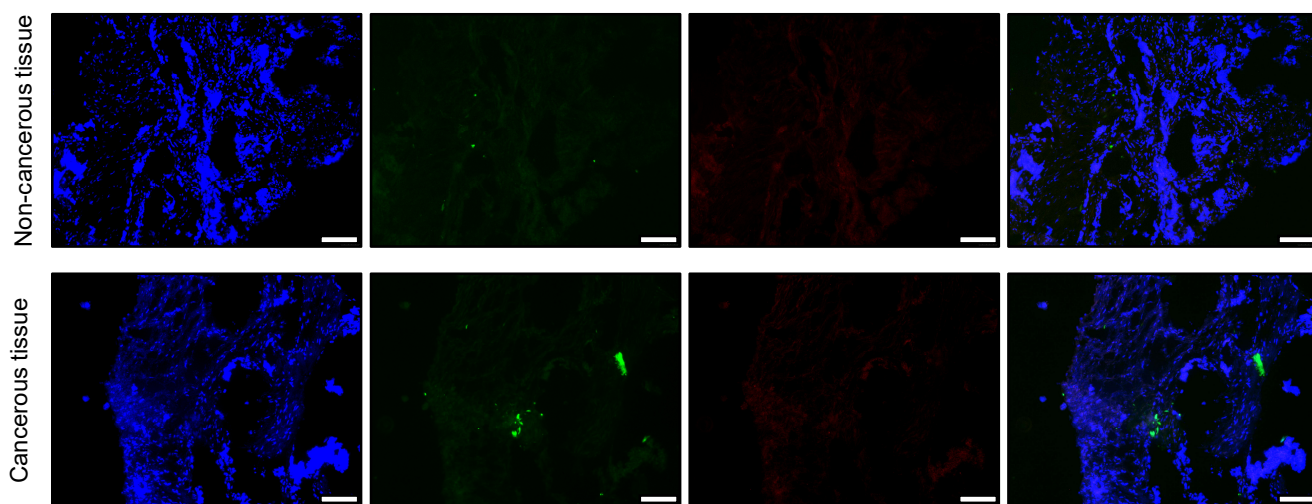

scale bars: 75 $\mu$ m

Supplement: Supplementary file 6 — Supplementary material [file MOL2-18-1278-s004.pdf]
