# Supplementary material for: Hypoxia‐induced miR‐210‐3p expression in lung adenocarcinoma potentiates tumor development by regulating CCL2 mediated monocyte infiltration
Source: Mol Oncol. 2024 Mar 22;18(5):1278–300. doi: 10.1002/1878-0261.13260 (PMC11077004; doi:10.1002/1878-0261.13260)
Supplement: Supplementary file 5 — Table S1. Clinical characteristics of the lung adenocarcinoma and noncancerous patients. Table S2. List of primers with sequence details. [file MOL2-18-1278-s005.pdf]

Supplementary Information

Hypoxia-induced miR-210-3p expression in lung adenocarcinoma potentiates tumor development by regulating CCL2-mediated monocyte infiltration

Leena Arora[a], Debarun Patra[a], Soumyajit Roy[a], Sidhanta Nanda[a], Navneet Singh[b], Anita K Verma[c], Anuradha Chakraborti[d], Suman Dasgupta[e], and Durba Pal[a*]

[a]Department of Biomedical Engineering, Indian Institute of Technology Ropar, Punjab 140001, India
[b]Department of Pulmonary Medicine, Postgraduate Institute of Medical Education & Research (PGIMER), Chandigarh 160012, India
[c]Department of Zoology, Kirori Mal College, University of Delhi, Delhi 110007, India
[d]Department of Experimental Medicine & Biotechnology, Postgraduate Institute of Medical Education & Research (PGIMER), Chandigarh 160012, India
[e]Department of Molecular Biology & Biotechnology, Tezpur University, Assam 784028, India

*Corresponding Author:
Durba Pal, PhD
Department of Biomedical Engineering,
Indian Institute of Technology Ropar,
Rupnagar, Punjab 140001, India.
E-mail: durba.pal@iitrpr.ac.in;
Phone: (+91) 01881-23-2506
ORCID: 0000-0001-7672-3529

Supplementary Tables

Table S1:

**Demographic and clinicopathological feature**	
**Lung adenocarcinoma (LUAD) patients**	
**Number of patients**	6
**Age**	
**Median(range)**	50 yr (38-75 yr)
**Gender**	
**Male**	3
**Female**	3
**Smoking history**	
**Smoker**	2
**Non-smoker**	4
**Histological subtype**	Lung adenocarcinoma
**Non-cancerous patients**	
**Number of patients**	6
**Age**	
**Median(range)**	54 yr (24-75 yr)
**Gender**	
**Male**	2
**Female**	4
**Smoking history**	
**Smoker**	1
**Non-smoker**	5

Table S2:

Gene name	Forward primer (5'-3')	Reverse primer (5'-3')
**Human Primers**		
CCL2	TCATAGCAGCCACCTTCATTC	CTCTGCACTGAGATCTTCCTATTG
HIF-1A	GTGGTGGTTACTCAGCACT	CGTCCCTCAACCTCTCAGTT
DNMT1	TACCTGGACGACCCTGACCTC	CGTTGGCATCAAAGATGGACA
DNMT3A	TATTGATGAGCGCACAAGAGAGC	GGGTGTTCCAGGGTAACATTGAG
DNMT3B	GGCAAGTTCTCCGAGGTCTCTG	TGGTACATGGCTTTTCGATAGGA
CD206	ACGATCCGACCCTTCCTTGA	GCTTGCAGTATGTCTCCGCT
CD163	AAAGCGAAGACAGAGACAGC	TCATGGGAATTTTCTGAGGAAT
CD80	CTCTTGGTGCTGGCTGGTCTTT	GCCAGTAGATGCGAGTTTGTGC
CD86	CCATCAGCTTGTCTGTTTCATTCC	GCTGTAATCCAAGGAATGTGGTC
iNOS	CTACCACACCCGAGATGGC	CCAAACACCAAGGTCATGCG
GAPDH	CCCTTCATTGACCTCAACTACA	ATGACAAGCTTCCCGTTCTC
Mutant CCL2-3'UTR	GACCCTCAAAACATCCCAGGGGT AGAAGACACCATCAAGAGGAAAA GCAATTTCCCCAAGT	ACTTGGGGAAATTGCTTTTCCTCTT GATGGTGTCTTCTACCCCTGGGATG TTTTGAGGGTC
**Zebrafish Primers**		
MPEG1.1	CGGGTTCAAGTCCGTAACCA	TGGCGTCAGCGATTTCTTCT
MPX	TCCGAGATGGCGATAGGTTG	AGGACTCCGGAAGCGAAAAG
β-Actin	AGAGCTATGAGCTGCCTGACG	CCGCAAGATTCCATACCCA
